# Prevention of STAT3-related pathway in SK-N-SH cells by natural product astaxanthin

**DOI:** 10.1186/s12906-023-04267-3

**Published:** 2023-11-29

**Authors:** Shao-Qian Sun, Feng-Xiang Du, Li-Hua Zhang, Hao-Shi, Fu-Ying Gu, Yu-Lin Deng, Yi-Zhi Ji

**Affiliations:** 1 School of Medical Technology, Beijing Institute of Technology, No. 5 Zhongguancun South Street, Haidian District, Beijing, 100081 China; 2 Biochemical Engineering College, Beijng Union University, Beijing, 100023 China

**Keywords:** Neuroblastoma, Astaxanthin, STAT3, Cell proliferation, Apoptosis, Cell migration inhibition, Cell invasion inhibition

## Abstract

**Purpose:**

Neuroblastoma (NB) is the most common solid malignancy in children. Despite current intensive treatment, the long-term event-free survival rate is less than 50% in these patients. Thus, patients with NB urgently need more valid treatment strategies. Previous research has shown that STAT3 may be an effective target in high-risk NB patients. However, there are no effective inhibitors in clinical evaluation with low toxicity and few side effects. Astaxanthin is a safe and natural anticancer product. In this study, we investigated whether astaxanthin could exert antitumor effects in the SK-N-SH neuroblastoma cancer cell line.

**Method:**

MTT and colony formation assays were used to determine the effect of astaxanthin on the proliferation and colony formation of SK-N-SH cells. Flow cytometry assays were used to detect the apoptosis of SK-N-SH cells. The migration and invasion ability of SK-N-SH cells were detected by migration and invasion assays. Western blot and RT-PCR were used to detect the protein and mRNA levels. Animal experiments were carried out and cell apoptosis in tissues were assessed using a TUNEL assay.

**Result:**

We confirmed that astaxanthin repressed proliferation, clone formation ability, migration and invasion and induced apoptosis in SK-N-SH cells through the STAT3 pathway. Furthermore, the highest inhibitory effect was observed when astaxanthin was combined with si-STAT3. The reason for this may be that the combination of astaxanthin and si-STAT3 can lower STAT3 expression further than astaxanthin or si-STAT3 alone.

**Conclusion:**

Astaxanthin can exert anti-tumor effect on SK-N-SH cells. The inhibitory effect was the higher when astaxanthin was combined with si-STAT3.

**Supplementary Information:**

The online version contains supplementary material available at 10.1186/s12906-023-04267-3.

## Introduction

Neuroblastoma (NB) is the most common solid malignancy in children [1] and accounts for 15% of the mortality rate of children’s tumors [2, 3]. NB is formed by the abnormal development of primitive neural crest cells, which form sympathetic ganglia during the embryonic stage. Because of their clinical features, such as hidden sites, difficult early diagnosis, high malignancy, strong invasion and rapid progression, the prognosis of NB tumors is poor and the 5-year survival rate is less than 40%. In addition, more than 75% of older children have tumors that metastasize to the bone marrow, liver and other places through lymphatic or blood routes [4]. At present, the treatment for NB mainly includes multidisciplinary combined treatment including large doses of chemical drugs, surgery, radiotherapy, stem cell transplantation and biological immunotherapy [5, 6]. Despite this intensive treatment, the long-term event-free survival rate of these patients is less than 50% [5]. Thus, patients with NB urgently need more valid treatment strategies.

STAT3, which belongs to the STAT family, is a gene located on the long arm of human chromosome 17. The STAT3 protein is composed of 750 ~ 850 amino acids and has dual functions of signal transduction and transcriptional regulation. It mediates extracellular signal transcription in the nucleus [7]. STAT3 regulates the secretion of cytokines by tumor cells in addition to the cell cycle process and apoptosis. It regulates tumor trophoblastic angiogenesis and the immune response of interstitial cells [7]. Research has found that the expression of STAT3 is abnormally high and persistently activated in many human solid tumors and hematoma cells [8], such as thyroid carcinoma [9], liver carcinoma [10], breast carcinoma [11] and pancreatic carcinoma [12]. Inhibiting the activation of STAT3 using knockout systems or inhibitors can significantly repress tumor progression, emphasizing the importance of blocking the STAT3 signal cascade in cancer therapy.

Previous scientific studies have suggested that inhibiting the expression of STAT3 can activate the apoptotic machinery, decrease neuroblastoma tumorigenicity, increase chemosensitivity and exert antimetastatic effects on neuroblastoma cells [13–15]. The overexpression of STAT3 is closely related to the tumorigenicity of neuroblastoma [13, 14, 16–18]. This suggests that STAT3 may be an available target for neuroblastoma.

STAT3 is a key oncogene and an important tumor target, and scholars have carried out a considerable amount of research to develop STAT3 inhibitors. Although many small molecules of STAT3 are currently undergoing many preclinical and clinical trial [12], no small molecule inhibitor that directly inhibits STAT3 has acquired the approval as a clinical drug by the US Food and Drug Administration. There are two mainly reasons: the limited clinical efficacy and the toxic side effect [19, 20]. Thus, STAT3 inhibitors with good clinical efficacy and fewer side effects need to be found urgently [19, 21, 22].

Astaxanthin is a safe and natural product [23] and one of the most common carotenoids. It is primarily found in shrimp, crab and salmon [24]. In 1987, astaxanthin was approved as a feed additive in the aquaculture industry by the United States Food and Drug Administration (USFDA). In 1999, astaxanthin was approved for use as a dietary supplement (nutraceutical) [25]. It has strong powerful antioxidative effects in all kinds of oxidative stress disease models [26, 27]. Recently, many in vivo and in vitro studies have shown that astaxanthin has antitumor effects against colon carcinogenesis, oral cancer, leukemia and hepatocellular carcinoma [28]. The mechanism behind the antitumor effects exerted by astaxanthin may be mediated by JAK-2/STAT3, ERK, AKT, NF-κB, PPARγ and Nrf2 [28]. However, whether neuroblastoma can be inhibited by astaxanthin through the STAT3 pathway has yet to be explored.

Here, we assessed whether astaxanthin could effectively inhibit the proliferation, clone formation, invasion and migration of neuroblastoma cells and increase apoptosis through the STAT3 pathway. We also examined whether the combination of astaxanthin and si-STAT3 had a better inhibitory effect.

## Materials and methods

### Cell culture and reagents

The SK-N-SH cells were purchased from the Cell Resource Center, Peking Union Medical College (the headquarters of the National Science & Technology Infrastructure, National BioMedical Cell-Line Resource, NSTI-BMCR). The cells were cultured in DMEM-H medium (M&C Gene Technology, Beijing, China) with 10% fetal bovine serum (FBS, Gibco, Auckland, New Zealand). The SK-N-SH cells were cultured in incubator with 5% CO_2_ at 37 °C. Astaxanthin was obtained from Sigma-Aldrich (St. Louis, MO, USA).

### MTT assay

SK-N-SH cells were prepared in a single cell suspension and inoculated into a 96-well plate at a volume of 200µL per well (5000 cells per well). The cells were cultivated for 72 h with different concentrations (0, 50, 100, 200 and 400 µM) of drugs in 100µL of medium. Then, 20µL of MTT solution was added. After 4 h, cultivation was stopped. The culture medium from each well was carefully absorbed and discarded. Each well was then shaken for 10 min with 150ul of DMSO decolorization shaker to ensure the crystals fully dissolved. The absorption value of each well was measured using an ELISA reader (Cayman Chemical, Ann Arbor, MI, USA) at a wavelength of 490 nm and the results were recorded.

### Colony formation assay

Treatment: The experiment was divided into five groups: CK, si-NC (siRNA-NC), astaxanthin, si-STAT3 and astaxanthin + si-STAT3. The si-NC(siRNA-NC) and si-STAT3 groups were separately transfected with an siRNA-NC plasmid or si-STAT3, respectively, for 48 h. Then, all the groups were given astaxanthin (200 µ m) for 24 h.

Clone formation experiment: After 14 days of culture, the culture media were discarded. The cells were rinsed twice with PBS. After 4% paraformaldehyde fixation for 20 min, two PBS rinses, 0.1% crystal violet staining for 20 min, another two PBS rinses and drying, the photograph was taken.

### Flow cytometry assay

Apoptosis was detected using an Annexin V-FITC/PI assay. Cells were digested with trypsin, centrifuged and collected. The cells were then washed with PBS twice and centrifuged at 4 °C for 5 min; a total of 1*10^5^ cells were collected. The cells were resuspended in 100 µL of buffer and 5 µL FITC and 5 µL PI stain solution were added. Following incubation for 5 min in the dark, the cells were supplemented with 500 µL of buffer and apoptosis was checked and measured using flow cytometry (Columbus 2.4, PerkinElmer, Waltham, MA, USA).

### Cell migration and cell invasion assay

A migration transwell with two chambers was used to detect the migration of the SK-N-SH cells. The SK-N-SH cells from each group were collected and resuspended in 2% serum medium. Then, the cells were inoculated into the upper chamber, while culture medium with 10% serum was added to the lower chamber. After 16 h, the SK-N-SH cells were fixed with 4% paraformaldehyde. The cells in the upper layer of the lower chamber membrane were wiped off, stained with crystal violet for 20 min, washed with PBS twice, dried and photographed.

An invasion transwell with two chambers was used to detect cell invasion. The upper chamber was pretreated with Matrigel. Then, the cells were resuspended in 2% serum medium and inoculated into the upper chamber. Culture medium with 10% serum was added to the lower chamber. After 16 h, the SK-N-SH cells were fixed with 4% paraformaldehyde. The cells in the upper layer of the lower chamber membrane were wiped off, stained with crystal violet for 20 min, washed with PBS twice, dried and photographed.

### Western blot

Cells from each of the five groups were placed in a culture dish and cultivated for 24 h, and protein was extracted after drug treatment. Protein concentrations were checked and measured using a BCA protein quantitative kit (Tiangen, Beijing, China; PA102). An equivalent of 30 µg protein was collected from each group, boiled for 15 min, loaded onto SDS-PAGE and then transferred onto a PVDF membrane. The PVDF membrane was blocked with milk for 1.5 h, rinsed with PBS 3 times and then incubated with antibodies diluted to 1:1000 or 1:500 overnight at 4 °C. The primary antibodies used included anti-Bax (1:1000, ab7977, Abcam, USA), anti-Bcl-2 (1:1000, ab692, Abcam), anti-Caspase-3 (1:1000, ab32351, Abcam), anti-Caspase-9 (1:1000, ab32539, Abcam), anti-NF-kβp65 (1:1000, ab76302, Abcam), anti-JAK2 (1:1000, ab92552, Abcam), anti-Stat3 (1:1000, ab119352, Abcam), and anti-β-actin (1:1000, ab8227, Abcam).

Following primary antibody incubation, the PVDF membranes were incubated with the diluted 1:1000 secondary antibodies for 2 h and rinsed with PBS three times. The internal reference was β-actin.

### RT-PCR

Cells from the five groups were placed in a culture dish and incubated for 24 h. Total RNA was collected using Baosai lysis reagent (Baosai, Hangzhou, China; RE02050). Reverse transcription was carried out with the extracted total RNA from the cells (1 µg) using a Revert Aid First Strand cDNA synthesis kit (Baosai; RT02020). A fluorescence quantification kit (Baosai;Å PM10003) was used in qRT-PCR, where 40 g of cDNA was used as the template.

The primer sequences were as follows: Caspase3-F: ATCCAGTCGCTTTGTGCCAT; Caspase3-R: TTCTGTTGCCACCTTTCGGT; Bcl2-F: GCCTTCTTTGAGTTCGGTG; Bcl2-R: AGTCATCCACAGGGCGAT; Casp9-F: TGGGCTCACTCTGAAGACCT; Casp9-R: AGCAACCAGGCATCTGTTTA; NF-κB-F: GTTTCCGTTACAAGTGCGAG; NF-κB-R: TTGGGTGCGTCTTAGTGGTA; JAK2-F: AGTAAAGATGCCTTCTGGTGAA; JAK2-R: TCCATTTCCAAGTTCTCCACT; STAT3-F: AATACCATTGACCTGCCGATGT; STAT3-R: GGGTTCAGCACCTTCACCAT; GAPDH-F: TTTGGTATCGTGGAAGGACT; GAPDH-R: GAGGCAGGGATGATGTTCT.

### Construction of SK-N-SH tumor model

A total of thirty nude mice were obtained from Sibeifu Biotechnology Co., Ltd. Lethal by inhalation of carbon dioxide, and were randomly divided into five groups as follows:

Group A: Blank control (gastric saline infusion).

Group B: Blank transfection group (transfected with a blank vector).

Group C: Nude mice were given astaxanthin once a day at a concentration of 200 mg/kg. The weight of the nude mice was about 20 g and the astaxanthin concentration was 200 mg/ml.

Group D: Intervention group.

Group E: Intervention combined with intragastric astaxanthin administration.

For the construction of the SK-N-SH tumor model, the nude mouse was injected with 2 × 10^7^ logarithmic-phase SK-N-SH cells in the left armpit. Two weeks after inoculation, tumor-bearing nude mice underwent different treatments based on their groups.

After 4 weeks of continuous intragastric administration, the nude mice were killed and tumor tissues were extracted and weighed for the experiments.

### Ethics statement

All experiments were conducted strictly according to the institutional guidelines for animal research and were approved by the Committee on the Ethics of Animal Experiments of Beijing Union University (CIHFBUU-ZYZDS-B01-15-01).

### TUNEL assay

Paraffin sections were dewaxed with distilled water and washed with PBS 3 times for 5 min. Then, the sections were treated with proteinase K working solution at 37℃ for 30 min, washed with PBS 3 times for 5 min, treated with H_2_O_2_ at room temperature for 10 min and washed with PBS 3 times for 5 min. TdT enzyme reaction solution was added at 37℃ for 60 min in the dark, then the sections were washed with PBS 3 times for 5 min. The sections were treated with Streptavidin-HRP solution at 37 ℃ for 30 min in the dark and washed with PBS 3 times for 5 min. Following DAB color rendering, three five-minute PBS washes and post-treatment hematoxylin sealing, open field panoramic scanning was carried out.

### Statistical analysis

A Student’s t-test was used to determine whether there was a statistically significant difference between the two groups. P < 0.05 indicated statistical significance.

## Results

### Astaxanthin inhibits the proliferation of SK-N-SH cells

**Fig. 1:**
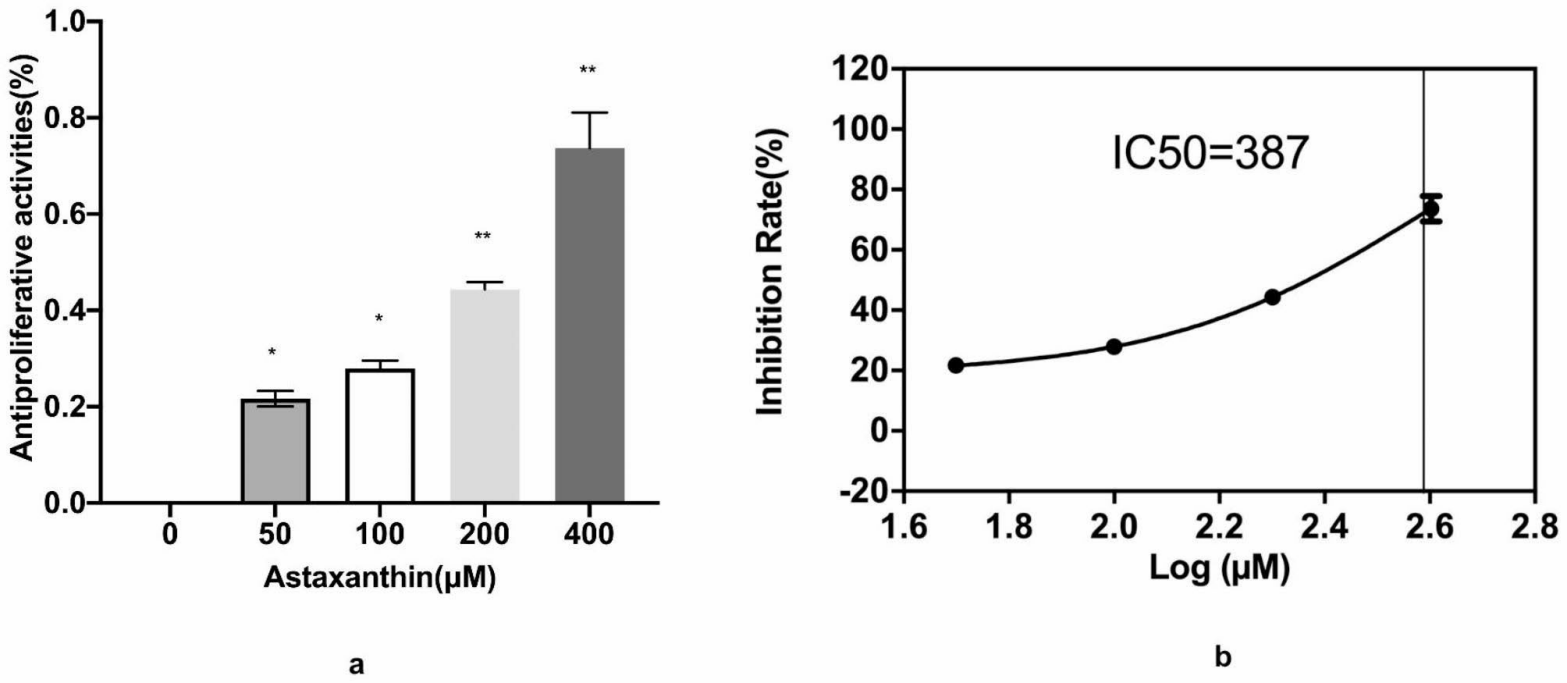
Antiproliferative effect of astaxanthin on SK-N-SH cells was studied using an MTT assay. (**a**) SK-N-SH cells were treated with 0, 50, 100, 200 and 400 µM astaxanthin for 48 h before the MTT assay was conducted. The data from three experiments were used to calculate mean values (± SD). *P < 0.05, **P < 0.01, ***P < 0.001. (**b**) Linear regression relationship between drug concentration and inhibition rate. IC50 was calculated as 387 using graphpad prism9

One of the most important features of tumor cells is their viability. An MTT assay was used to detect the inhibition of growth with astaxanthin treatment in human neuroblastoma SK-N-SH cells. The SK-N-SH cells were treated with astaxanthin at concentrations of 50, 100, 200 and 400µM for 72 h. The MTT assay was used to check the cell viability after treatment. The results of the MTT assay showed that astaxanthin significantly inhibited the proliferation of SK-N-SH cells in a concentration-dependent manner. Astaxanthin clearly repressed the proliferation of SK-N-SH cells at concentrations of 50, 100, 200 and 400µM. The inhibition rate was 21.7%, 27.9%, 44.4% and 73.7%, respectively (Fig. 1a).

According to the liner regression relationship between drug concentration and inhibition rate (Fig. 1b), IC50 was calculated as 387 µM using graphpad prism9. Nearly half of the IC50 (200 µM), was used as the concentration for the subsequent experiment.

### Astaxanthin suppresses the clone formation ability of SK-N-SH cells and shows a higher inhibition rate when combined with si-STAT3

Five groups were defined in this study: CK, si-NC (siRNA-NC), astaxanthin, si-STAT3 and astaxanthin + si-STAT3. CK was the blank control group. The si-STAT3 and si-NC (siRNA-NC) groups were transfected with siRNA-STAT3 and a blank siRNA-NC plasmid, respectively, for 48 h. Astaxanthin (200 µM) was administered for 24 h after transfection for 48 h.

**Fig. 2:**
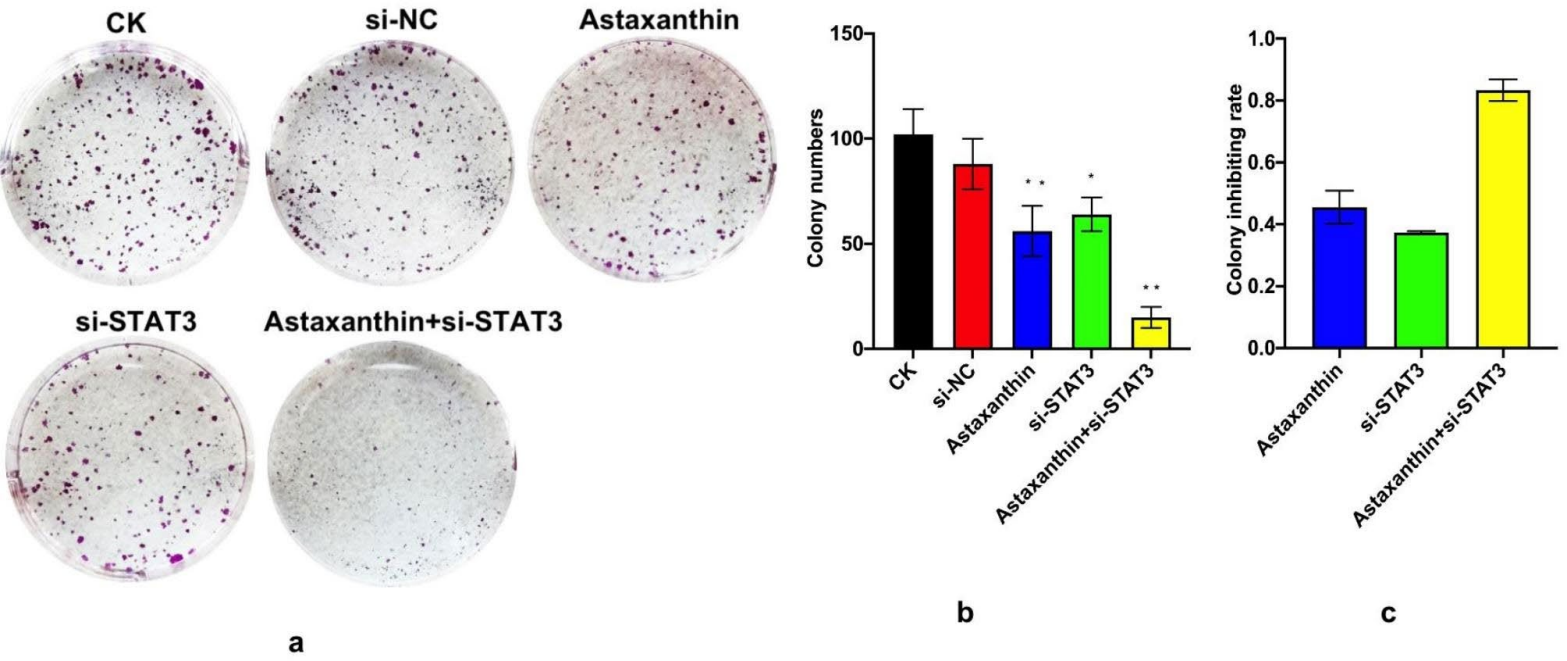
Clone formation assay. There were five groups in the experiment: CK, si-NC (siRNA-NC), astaxanthin, si-STAT3 and astaxanthin + si-STAT3. CK was the blank control group. The si-STAT3 and si-NC (siRNA-NC) groups were, respectively, transfected with siRNA-STAT3 and a blank siRNA-NC plasmid for 48 h. Astaxanthin (200 µM) was administered for 24 h after transfection for 48 h. (**a**) Photograph of the colonies formed in the five groups. (**b**) Results for clone formation number. The data are presented as the mean of three experiments (± SD). *P < 0.05, **P < 0.01, ***P < 0.001. (**c**) Results for clone formation inhibition rate. The data are presented as the mean of three experiments (± SD). *P < 0.05, **P < 0.01, ***P < 0.001

The results of the clone formation assay showed that astaxanthin, si-STAT3 and astaxanthin + si-STAT3 could all reduce the number of SK-N-SH cell colonies (Fig. 2a and b). The astaxanthin, si-STAT3 and astaxanthin + si-STAT3 groups had less clones compared to the NC and si-NC groups. The clone formation inhibition rate in the astaxanthin, si-STAT3 and astaxanthin + si-STAT3 groups was 45%, 37%, and 80%, respectively. Therefore, both astaxanthin and si-STAT3 effectively inhibited the cloning ability of SK-N-SH cells, where astaxanthin + si-STAT3 had the highest inhibition effect, reaching 83% (Fig. 2c).

### Astaxanthin induces apoptosis in SK-N-SH cells and shows a higher apoptosis rate when combined with si-STAT3

**Fig. 3:**
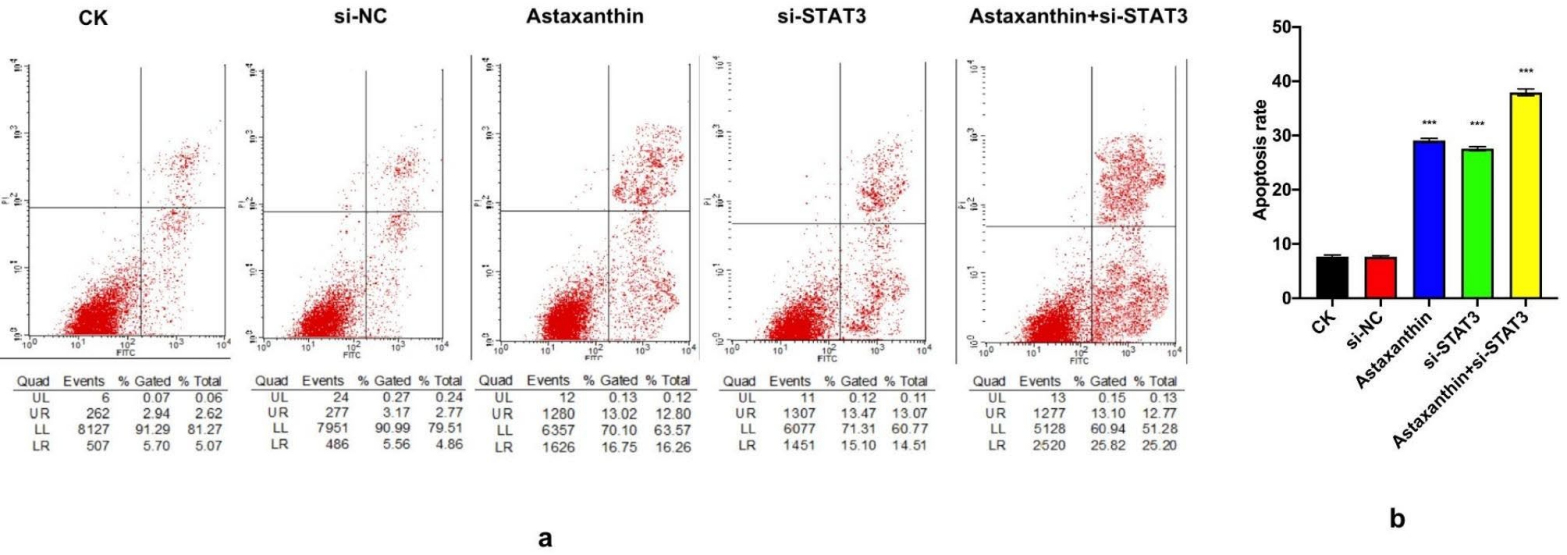
Determination of apoptosis rate. There were five groups in the experiment: CK, si-NC (siRNA-NC), astaxanthin, si-STAT3 and astaxanthin + si-STAT3. CK was the blank control group. The si-STAT3 and si-NC (siRNA-NC) groups were transfected with siRNA-STAT3 and a blank siRNA-NC plasmid, respectively, for 48 h. Astaxanthin (200 µM) was administered for 24 h following transfection for 48 h. (**a**) Detection of apoptosis using flow cytometry. (**b**) Apoptosis rate results. Silencing STAT3 increased the percentage of apoptotic cells. The group treated with astaxanthin combined with si-STAT3 showed the highest apoptosis rate. The data from three experiments were used to calculate mean values (± SD). Quad, quadrant; UL, upper left; UR, upper right; LL, lower left; LR, lower right. *P < 0.05, **P < 0.01, ***P < 0.001

Annexin V-fluorescein and PI double staining assays were used to determine the rate of apoptosis in SK-N-SH cells. Similarly, there were five groups in this experiment: a blank control (CK), astaxanthin, a blank control to si-STAT3 group (si-STAT3), si-STAT3 and astaxanthin + si-STAT3 (Fig. 3a). All groups were treated with astaxanthin for 24 h.

The results indicated that the apoptosis rates in the CK, si-NC, astaxanthin, si-STAT3 and astaxanthin + si-STAT3 groups were 7.6%, 7.3%, 29%, 28% and 38%, respectively (Fig. 3b), demonstrating that astaxanthin improved the apoptosis rate from 7.6 to 29%. Silencing STAT3 also increased the percentage of apoptosis from 7.3 to 28%. However, the group treated with astaxanthin combined with si-STAT3 showed the highest apoptosis rate, reaching 38%.

### Astaxanthin suppresses the migration and invasion of SK-N-SH cells and shows the best effect when combined with si-STAT3

**Fig. 4:**
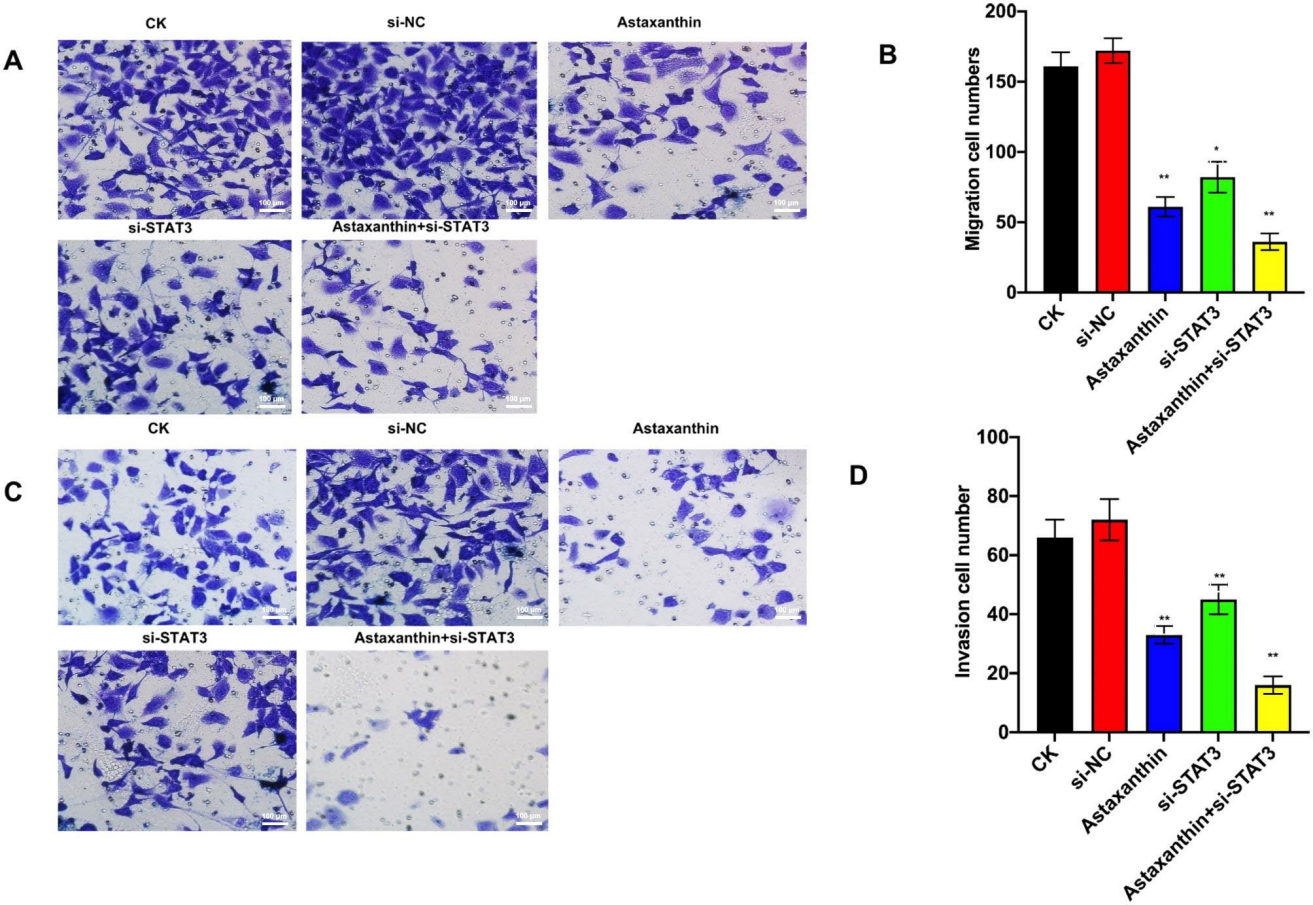
Migration and invasion of SK-N-SH cells. The concentration of astaxanthin used in the experiment was 200 µM/L. (**a**) Results of the migration assay. (**b**) Statistics for tumor cell migration in each group. Astaxanthin and si-STAT3 both decreased migration. However, astaxanthin combined with STAT3 showed the best effect. The data from three experiments were used to calculate mean values (± SD). *P < 0.05, **P < 0.01, ***P < 0.001. (**c**) Results of the invasion experiments. (**d**) Statistics for tumor cell invasion in each group. Both astaxanthin and si-STAT3 decreased invasion. However, astaxanthin combined with STAT3 showed the best effect. The data from three experiments were used to calculate mean values (± SD). *P < 0.05, **P < 0.01, ***P < 0.001

Migration and invasion are two of the most critical features of tumor cells. We conducted migration and invasion experiments in SK-N-SH cells. The concentration of astaxanthin was 200 µM/L. As shown in Fig. 4, astaxanthin and si-STAT3 both decreased migration and invasion. However, astaxanthin combined with STAT3 showed the best effect. The results showed that about 62% of cells failed to pass from the upper chamber to the lower chamber when treated with astaxanthin (P < 0.01) and 50% of cells failed to pass through the transwell membrane compared to the blank group. The inhibition rates for migration and invasion in the si-STAT3 group were 52% and 37%, respectively. However, the inhibition rates were improved to 79% and 78% when astaxanthin and si-STAT3 were combined.

### Astaxanthin inhibits SK-N-SH cells through a STAT3-related pathway, and shows the best effect when combined with si-STAT3

**Fig. 5:**
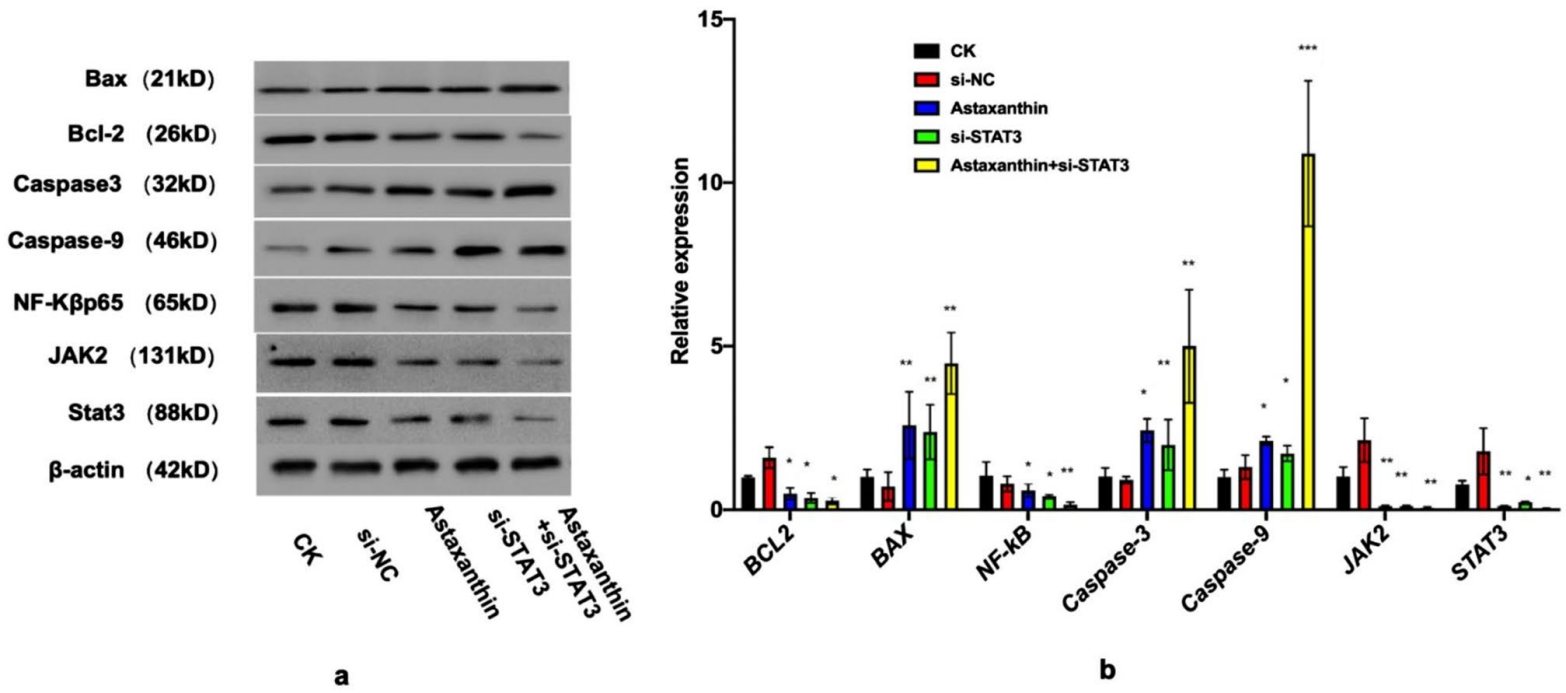
Astaxanthin inhibits the signal transduction pathways of STAT3 in SK-N-SH cells and shows a greater effect when combined with si-STAT3. (**a**) Western blot results. When SK-N-SH cells were treated with astaxanthin, the expressions of Caspase3, Caspase9 and Bax were all upregulated, while the expressions of JAK2, BCL-2, NF-кB and STAT3 were all downregulated. The full blots and additional explanations are shown in the Supplementary File 1. (**b**) RT-PCR result. The RT-PCR analyses were consistent with the results of the protein experiments

It has been shown that BAX, BCL-2, Caspase3, Caspase9, JAK2 and NF-кB are key proteins in STAT3-related pathways [29, 30]. In order to confirm if the inhibitory effects of astaxanthin were related to STAT3 pathways, we detected the expression of BAX, BCL-2, Caspase3, Caspase9, JAK2 and NF-кB. The results showed that when the SK-N-SH cells were treated with astaxanthin, the expressions of Caspase3, Caspase9 and BAX were all upregulated, while the expressions of BCL-2, NF-кB, JAK2 and STAT3 were all downregulated (Fig. 5a). Thus, astaxanthin can repress proliferation, promote apoptosis and suppress migration and invasion via STAT3-related pathways. As a consequence of the STAT3-inhibiting effect of astaxanthin, the expression of STAT3 was even lower when si-STAT3 was combined with astaxanthin, resulting the best inhibition effects in SK-N-SH cells.

RT-PCR (Fig. 5b) analyses were consistent with the results of the protein experiments. Astaxanthin increased the gene expression of Caspase3, Caspase9 and Bax and reduced the gene expression of Bcl-2, Jak2, Stat3 and Nf-кb.

These results confirmed that the repression of STAT3 and related genes by astaxanthin resulted in antitumor effects. The mRNA expression of stat3 was even lower when si-STAT3 was combined with astaxanthin, resulting in the best inhibition effect in SK-N-SH cells.

### Influence of astaxanthin and the combination of astaxanthin and si-STAT3 on the growth of xenograft tumors

Finally, we conducted animal experiments to explore whether astaxanthin and the combination of astaxanthin and si-STAT3 had inhibitory effects on xenograft tumors. We established five xenograft models in nude mice.

**Fig. 6:**
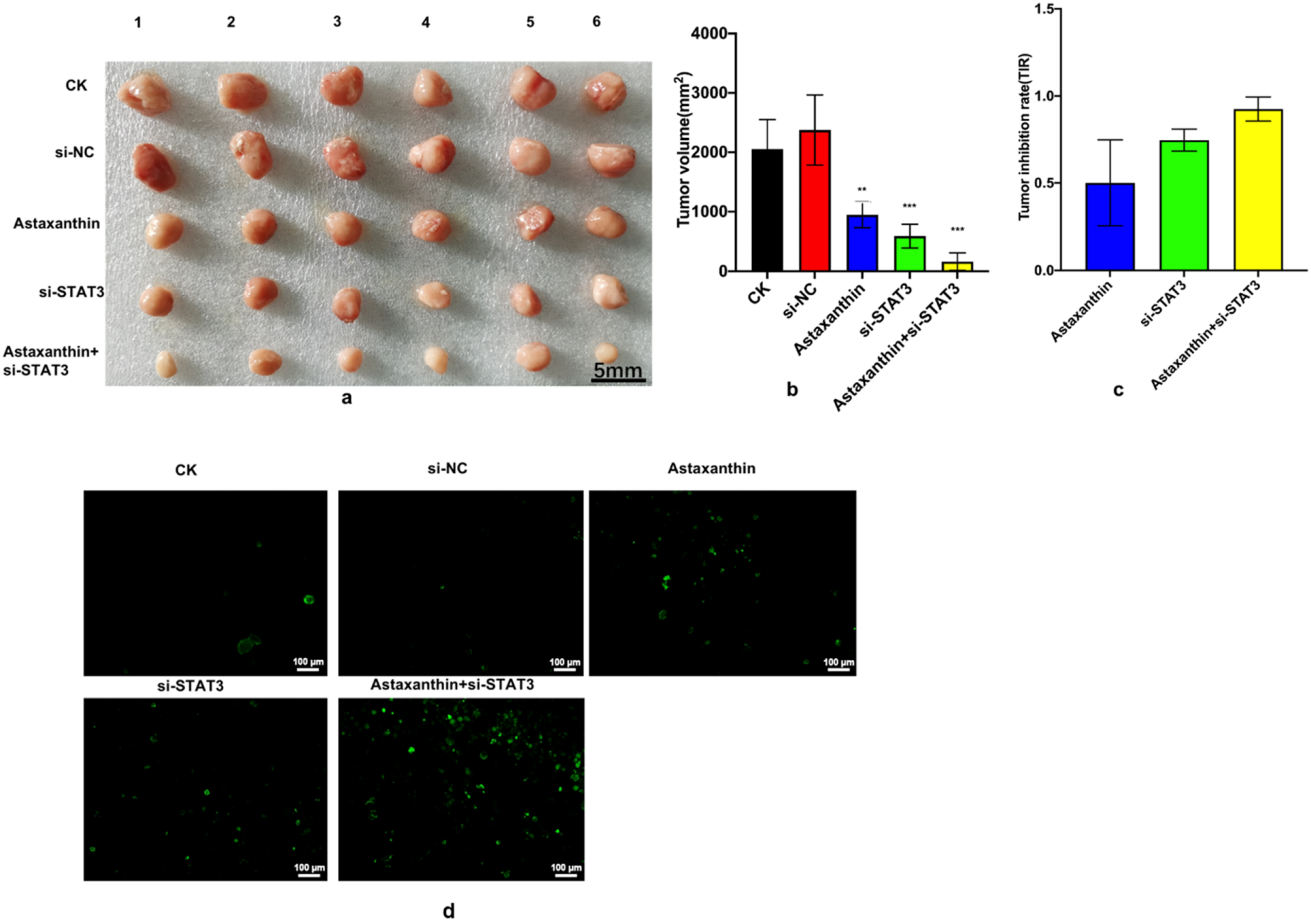
Influence of astaxanthin and the combination of astaxanthin and si-STAT3 on the growth of xenograft tumors. (**a**) Animal experiment results. Each group had six mice as parallel repeats. Numbers 1–6 represent the six mice used in the experiment to check the reproducibility of the results. (**b**) Animal experiment results. Astaxanthin and si-STAT3 both suppressed tumor growth. However, the repression effects were strengthened when astaxanthin and si-STAT3 were combined. The data from three experiments were used to calculate mean values (± SD). *P < 0.05, **P < 0.01, ***P < 0.001. (**c**) Results for tumor inhibition rate. (**d**) Cell apoptosis in tissues assessed using a TUNEL assay

The results showed that astaxanthin and si-STAT3 both suppressed the growth of tumors, with tumor volume reduction rates of 54% and 75%, respectively. However, this repression was strengthened when astaxanthin and si-STAT3 were combined, reaching 93% (Fig. 6a, b and c). The animal experiment results are consistent with those of the cell experiments. These results support the assumption that astaxanthin can significantly inhibit tumor growth through the STAT3 pathway; this effect was improved when astaxanthin and si-STAT3 were combined.

Thus, we can conclude that astaxanthin can suppress the expression of STAT3, leading to the repression of xenograft tumors. These results verify the hypothesis of the cell experiments.

In addition, a TUNEL assay (Fig. 6d) showed that astaxanthin and si-STAT3 both increased the number of apoptotic cells in tumor tissues. However, the number of the apoptotic cells in tissues was the highest when astaxanthin and si-STAT3 were combined.

## Discussion

Neuroblastoma (NB) is the most common solid malignancy in children, and more effective treatment strategies are urgently needed. It has been reported that STAT3 plays an important role in tumorigenicity and metastatic effects in neuroblastoma cells. Research has shown that astaxanthin can exert antitumor effects on tumor cells through the STAT3 pathway. However, whether astaxanthin can inhibit NB cells through the STAT3 pathway remains unknown. In this research, we demonstrated that astaxanthin can repress proliferation, cloning ability, migration and invasion and induce apoptosis in SK-N-SH cells through the STAT3 pathway. It was also found that this reduction was greatest when astaxanthin was combined with si-STAT3, which resulted in lower expression than either treatment alone. The reason may be the combination of astaxanthin and si-STAT3 can make STAT3 expression lower than that of single astaxanthin or single si-STAT3.

Recently, STAT3-targeted inhibitors have become a hot topic in the research of anti-tumor drugs. At the moment, there are two strategies to inhibit the STAT3 pathway: indirect inhibitors and direct inhibitors. Indirect inhibitors block the upstream molecules of the STAT3 pathway and inhibit the signal transduction function of STAT3 indirectly, for example, by repressing JAK and Src [31, 32]. Indirect inhibitors of STAT3 interact with these components to inhibit STAT3 activation and lead to the failure of STAT3 regulatory gene expression. However, although most of these inhibitors have entered into clinical use, they have not been well marketed. This is because most of these inhibitors are associated with adverse reactions and are difficult to be used as medicines [20]. Direct STAT3 inhibitors hinder the formation of functional STAT3 dimers by destroying the SH2DBD or NTD domains. However, some have failed to reach the standards of clinical trials due to a lack of stable membrane permeability and limited potential immunogenicity [20], while others fall short with respect to cell membrane permeability and valid targeted delivery vectors, hindering their application in tumors [33]. The other kind of direct inhibitors show good physicochemical properties in vitro, but most of them have poor clinical efficacy. Thus, STAT3 inhibitors with good clinical efficacy and fewer side effects urgently need to be found [20].

In this study, we confirmed that astaxanthin can exert antitumor effects via a STAT3-related pathway. Neuroblastoma (NB) is a serious type of tumor that affects children. Thus, in recent years, general and clinical research has been devoted to improving the survival rate of NB patients. Tumor metastasis and chemotherapy drug resistance in high-risk NB patients are difficult points in basic research and clinical treatment [5, 34]. How to improve the therapeutic efficiency of NB and reverse drug resistance has become an urgent problem to be addressed in the treatment of NB. It has been reported that STAT3 is also associated with resistance to conventional chemotherapy. Recent reports have shown that the inhibition of the STAT3 signaling pathway in drug-resistant cells can restore the chemotherapy effect, as tumor cells become resistant to platinum chemotherapy drugs by activating the STAT3 signaling pathway [35]. The results of this research showed that STAT3 activation was prevalent in chemotherapy-acquired drug resistance. The abnormal activation of STAT3 has been shown to be closely related to chemotherapy resistance in bladder cancer [36], blood cancer [37], glioma [38], ovarian cancer [39], breast cancer [40] and acute myeloid leukemia [41]. In addition, STAT3-siRNA and JAK2/STAT3 inhibitors have been shown to repress STAT3 activation and increase chemotherapy sensitivity [42, 43].

Astaxanthin, while generally recognized as safe in appropriate doses, may have side effects at higher concentrations [44]. In clinical trials, it’s crucial to determine the optimal dose that balances therapeutic effects with minimal toxicity. Moreover, the long-term effects of astaxanthin and STAT3 modulation are not yet fully understood. Clinical trials should incorporate long-term follow-up to assess the safety and efficacy of treatment over time. Perspectives include post-marketing surveillance and real-world evidence studies. Besides, astaxanthin may interact with certain medications, such as blood thinners and immunosuppressants, so it is important to consider the effect of combination application of astaxanthin and other drugs [45]. Therefore, whether astaxanthin can become a well-established therapeutic strategy in the treatment of neuroblastoma remains to be further studied.

**Fig. 7:**
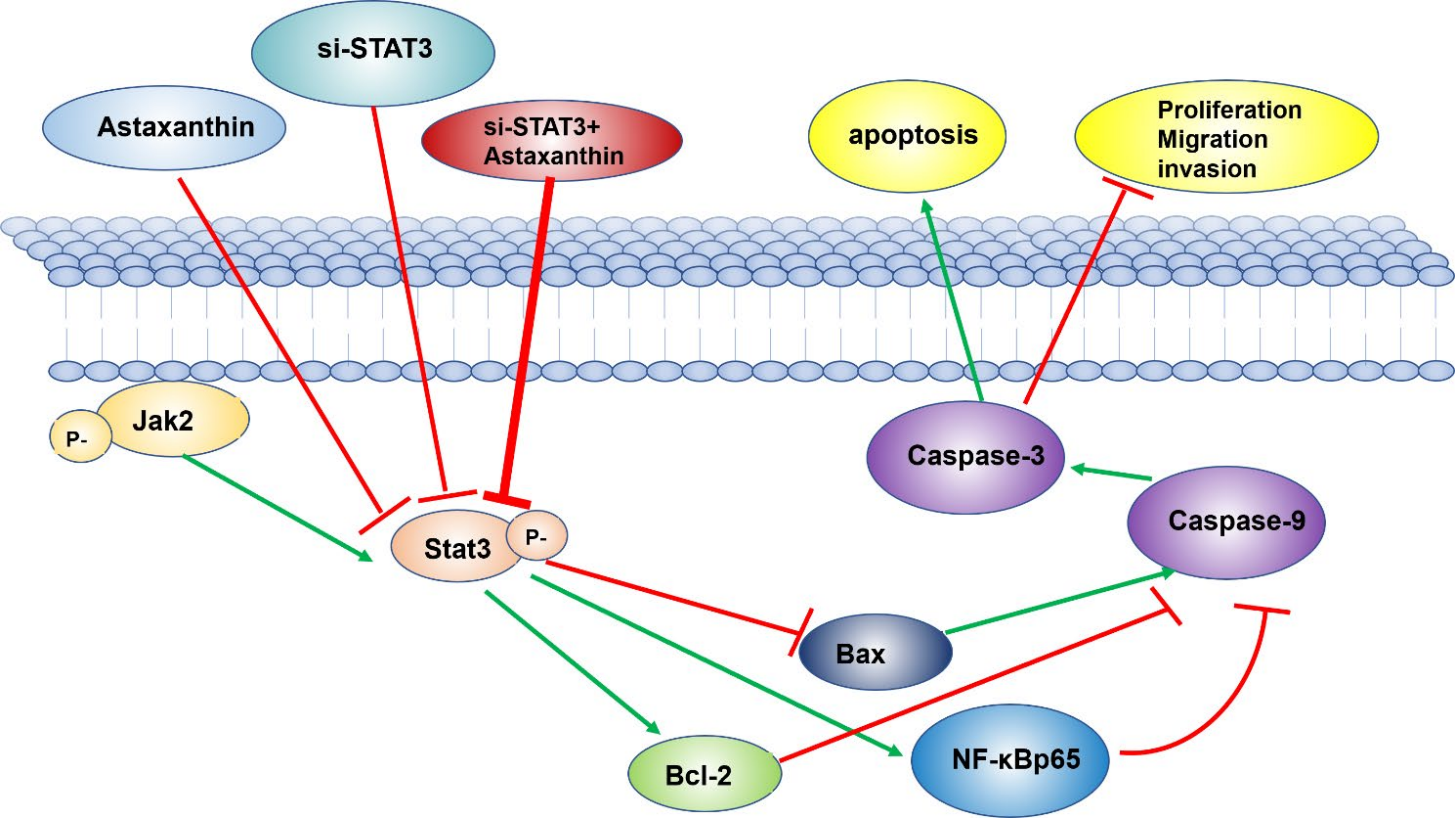
Mechanism of astaxanthin on neuroblastoma SK-N-SH cancer cells. Astaxanthin inhibits SK-N-SH tumor cells through a STAT3-related pathway. These effects are improved when astaxanthin and si-STAT3 are combined

In this research, we demonstrated that astaxanthin can improve the apoptosis rate in SK-N-SH cells by repressing metastasis through the STAT3 pathway (Fig. 7). However, whether astaxanthin can reverse drug resistance in SK-N-SH cells through the STAT3 pathway remains to be explored.

## Conclusions

Astaxanthin can repress proliferation, cloning ability, migration and invasion and induce apoptosis in SK-N-SH cells through the STAT3 pathway. We also found that this inhibitory effect was the highest when astaxanthin was combined with si-STAT3, as opposed to either treatment alone. However, whether astaxanthin is a direct or indirect inhibitor of STAT3 requires further research.

### Electronic supplementary material

Below is the link to the electronic supplementary material.

Supplementary Material 1: Full blots and additional explanations

## Data Availability

All data generated during this study are included in this published article.
