# Supplementary material for: Prevention of STAT3-related pathway in SK-N-SH cells by natural product astaxanthin
Source: BMC Complement Med Ther. 2023 Nov 29;23:430. doi: 10.1186/s12906-023-04267-3 (PMC10685499; doi:10.1186/s12906-023-04267-3)
Supplement: Supplementary file 1 — Supplementary Material 1: Full blots and additional explanations [file 12906_2023_4267_MOESM1_ESM.pdf]

(1) MTT assay

group	blank	Experimental group	Remove blank	Inhibiting rate
**CK**	0.102	1.359	1.257	0
	0.102	1.169	1.067	0
	0.112	0.921	0.809	0
**给药 50uM**	0.432	1.411	0.979	0.221
	0.401	1.223	0.822	0.23
	0.359	1.007	0.648	0.199

SK-N-SH	blank	Experimental group	Remove blank	Inhibiting rate
CK	0.102	1.359	1.257	0
	0.102	1.169	1.067	0
	0.112	0.921	0.809	0
给药 100uM	0.386	1.281	0.895	0.288
	0.461	1.219	0.758	0.29
	0.444	0.444	0.599	0.26

SK-N-SH	blank	Experimental group	Remove blank	Inhibiting rate
CK	0.102	1.359	1.257	0
	0.102	1.169	1.067	0
	0.112	0.921	0.809	0
给药 200uM	0.411	1.115	0.704	0.44
	0.483	1.059	0.576	0.46
	0.356	0.816	0.460	0.431

SK-N-SH	blank	Experimental group	Remove blank	Inhibiting rate
CK	0.102	1.359	1.257	0
	0.102	1.169	1.067	0
	0.112	0.921	0.809	0
给药 400uM	0.412	0.837	0.425	0.662
	0.496	0.776	0.280	0.738
	0.398	0.552	0.154	0.81

Group	Colony formation ability
CK	102±12

si-NC	88±12
astaxanthin	56±12
Si-STAT3	64±8
combined	15±5

Colony formation picture:

CK	Si-NC	astaxanthin
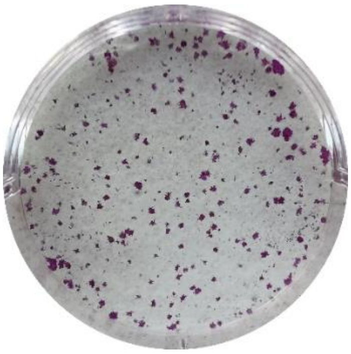	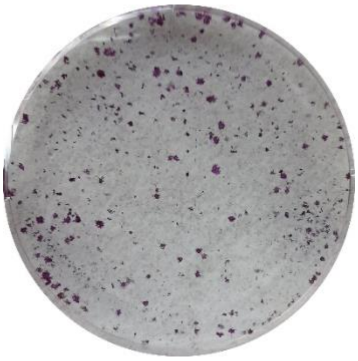	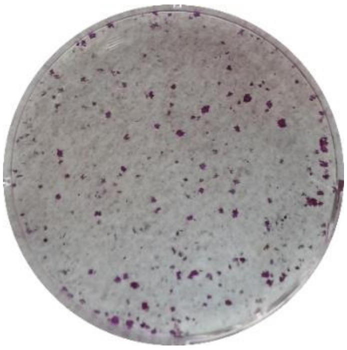
Si-stat3	combined	
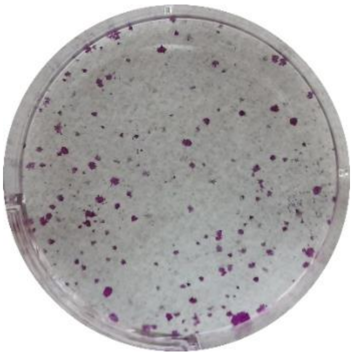	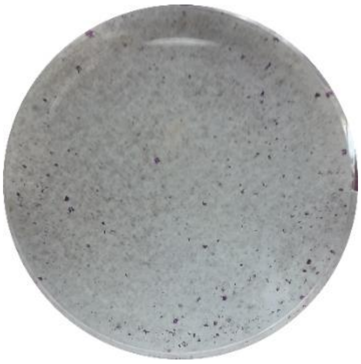	

migration assay picture

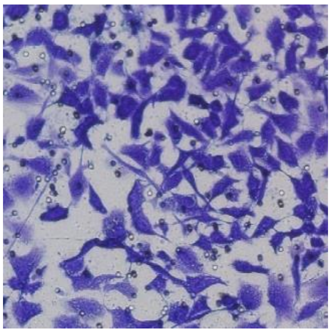

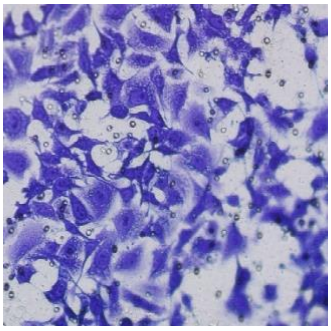

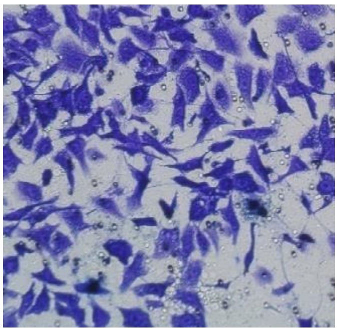

CK-1 CK-2 CK-3

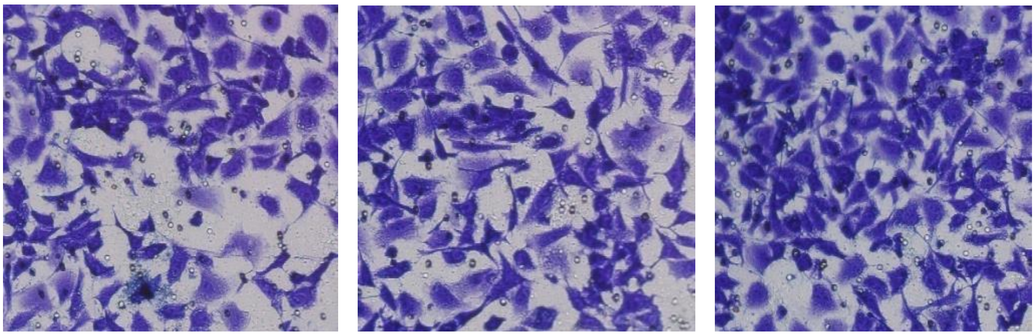

si-NC-1 si-NC-2 si-NC-3

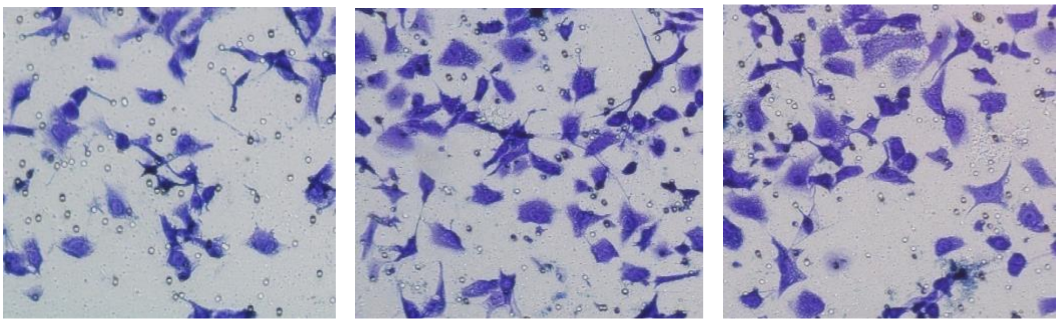

Astaxanthin-1 Astaxanthin-2 Astaxanthin-3

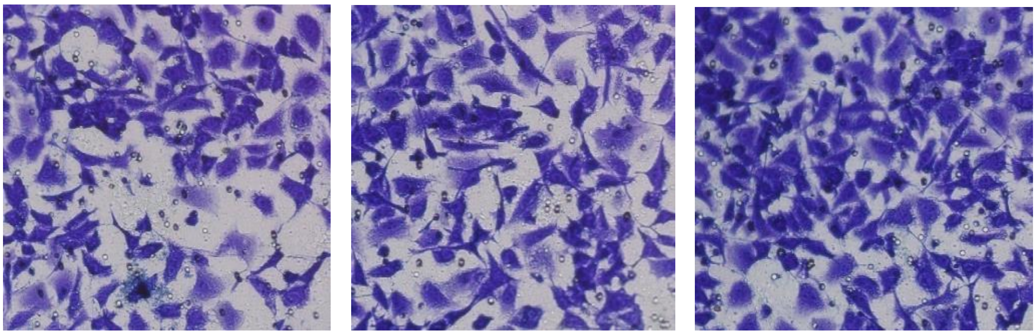

si-STAT3-1 si-STAT3-2 si-STAT3-3

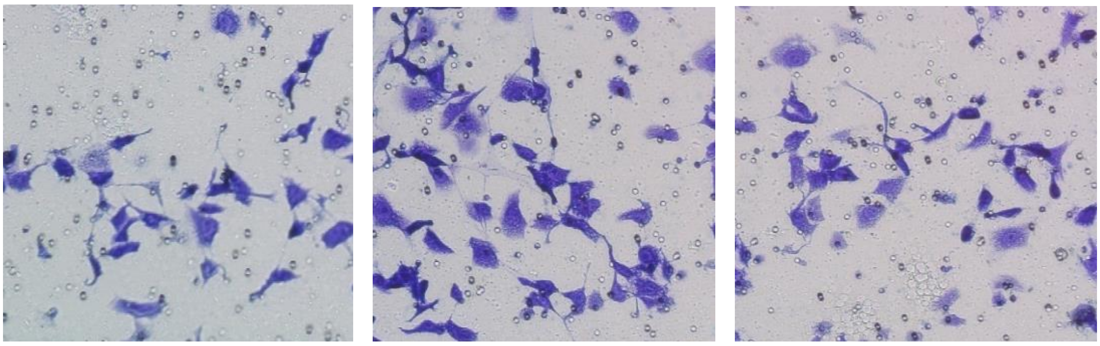

Combined-1 Combined-2 Combined-3

invasion assay picture

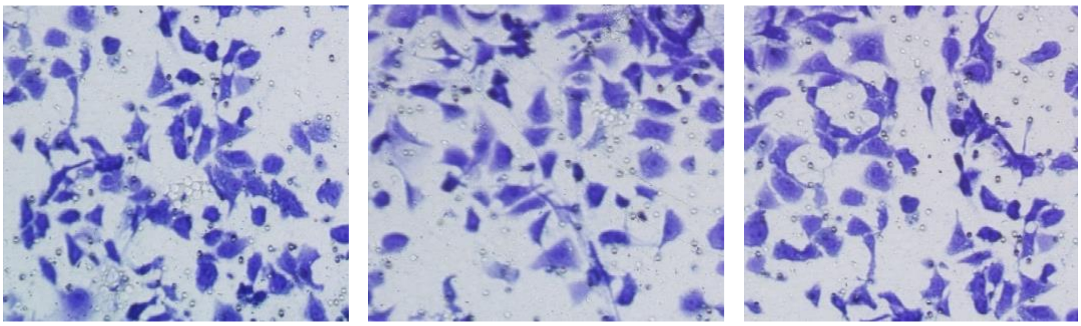

CK-1 CK-2 CK-3

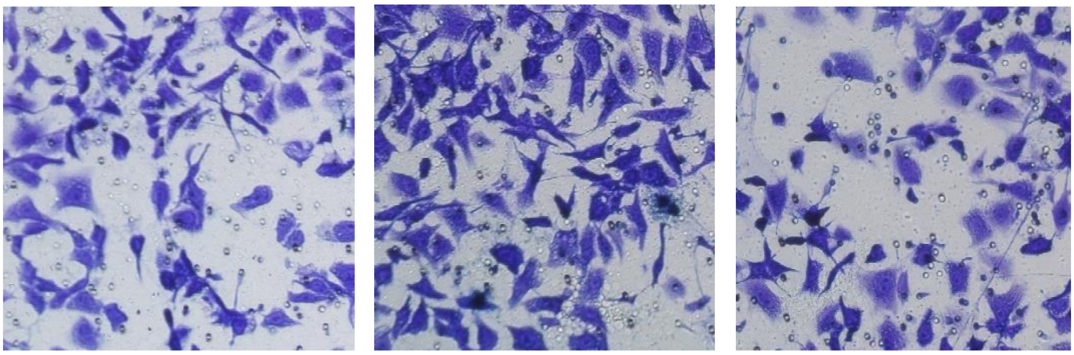

si-NC-1 si-NC-2 si-NC-3

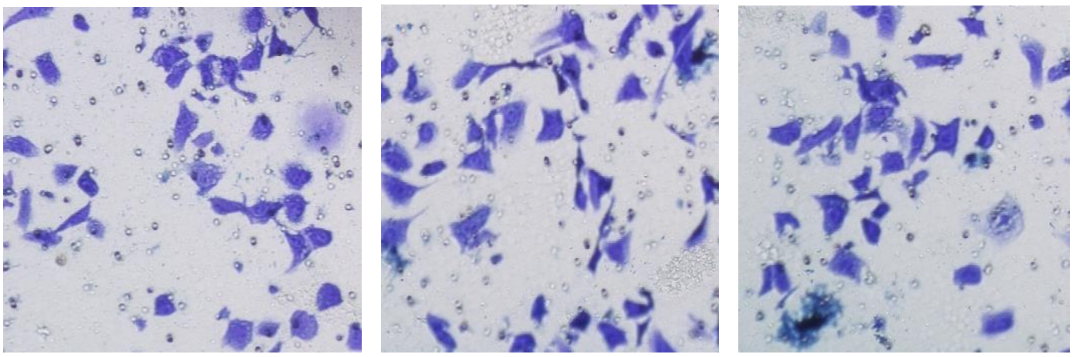

Astaxanthin-1 Astaxanthin-2 Astaxanthin-3

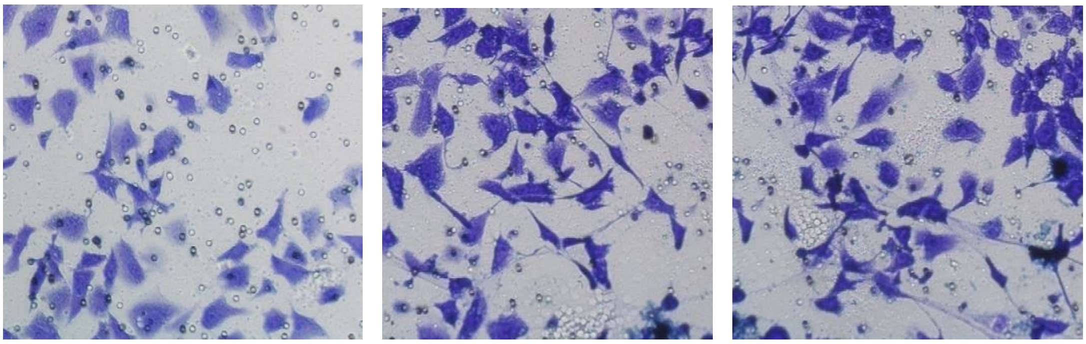

si-STAT3-1 si-STAT3-2 si-STAT3-3

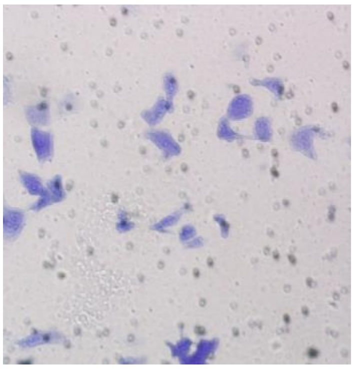

Combined-1

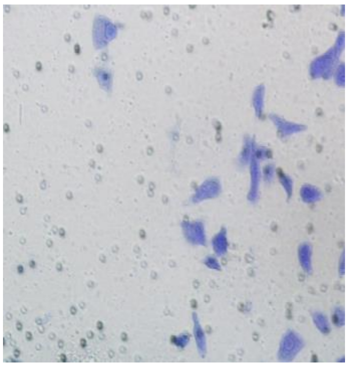

Combined-2

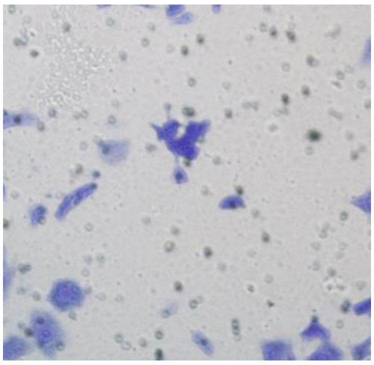

Combined-3

Apoptosis assay

Apoptosis assay：

组别	凋亡率%
CK	7.69±0.31
si-NC	7.63±0.25
astaxanthin	29.06±0.41
si-stat3	27.58±0.37
astaxanthin+si-stat3	37.97±0.62

凋亡图：

CK	NC	虾青素
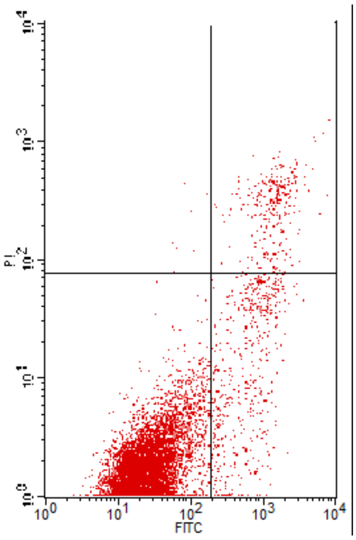	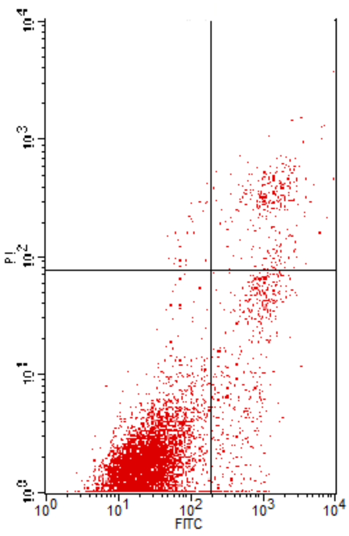	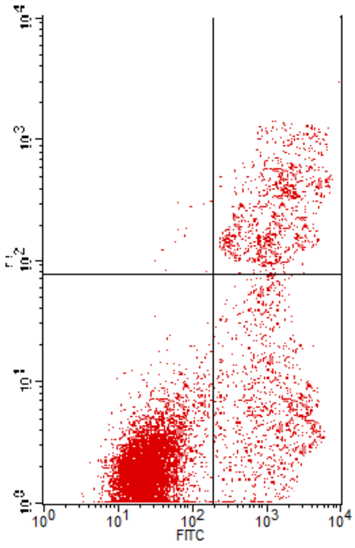
干扰	NC+EGF	

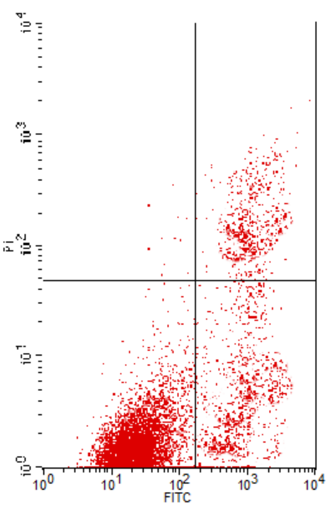	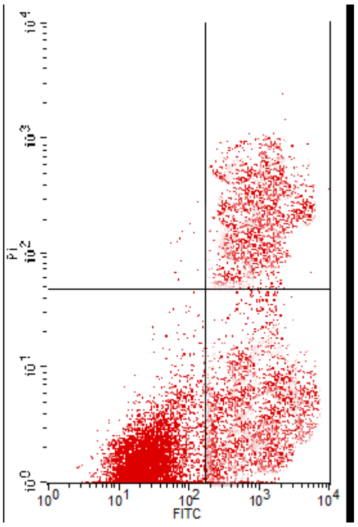	

RT-PCR data

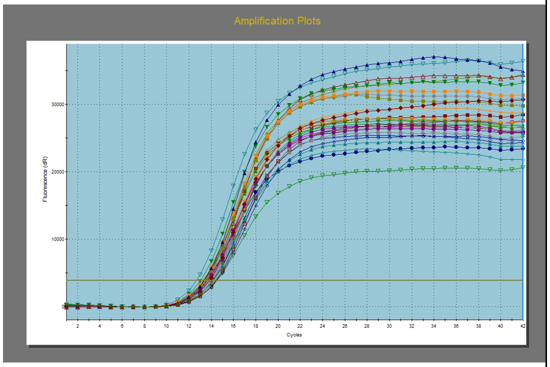 Actin 扩增曲线	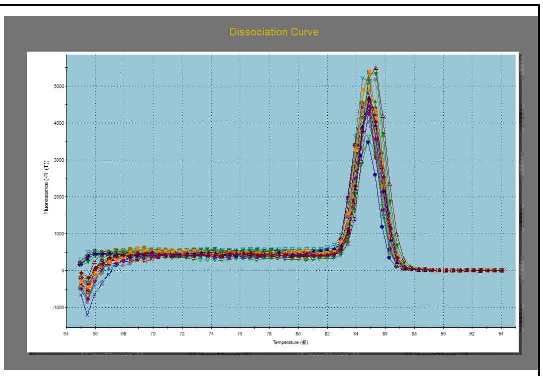 Actin 溶解曲线
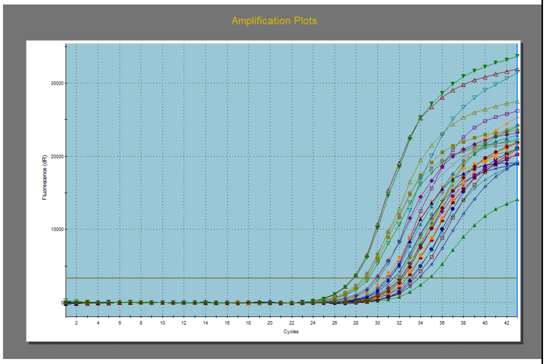 Bcl2 扩增曲线	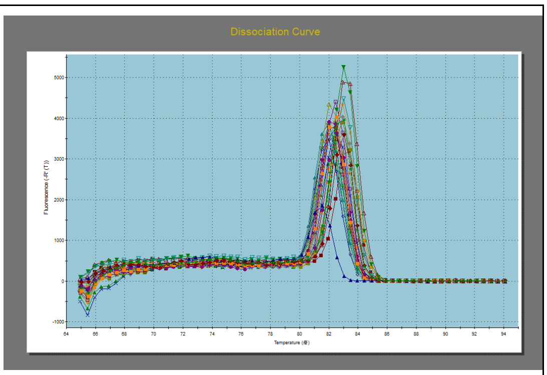 Bcl2 溶解曲线

扩增结果

BCL2	Ct (dR)	actin	Ct (dR)	△CT	△△CT	相对表达量

	30.95		13.74	17.21	0.02	0.98
ck	30.69	ck	13.45	17.24	0.05	0.96
	30.51		13.40	17.11	-0.08	1.05
	29.98		13.61	16.37	-0.82	1.76
si-NC	29.91	N	13.57	16.34	-0.85	1.80
	30.07		13.18	16.89	-0.30	1.23
	31.87		13.09	18.78	1.59	0.33
astaxanthin	31.85	E+G	13.64	18.21	1.02	0.49
	31.07		13.33	17.74	0.55	0.68
	33.22		13.72	19.50	2.31	0.20
si-stat3	32.76	I	14.55	18.21	1.02	0.49
	32.83		14.37	18.46	1.27	0.41
	32.16		12.73	19.43	2.24	0.21
combined	32.14	M+E	13.68	18.46	1.27	0.41
	32.61		13.32	19.29	2.10	0.23

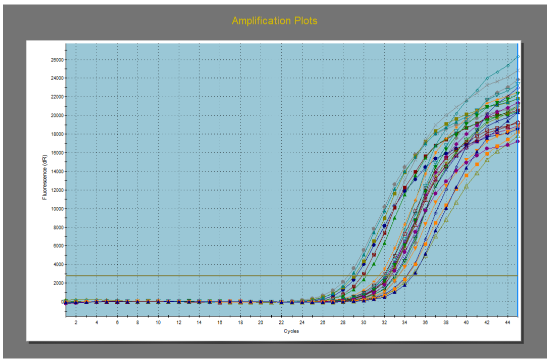

Bax 扩增曲线

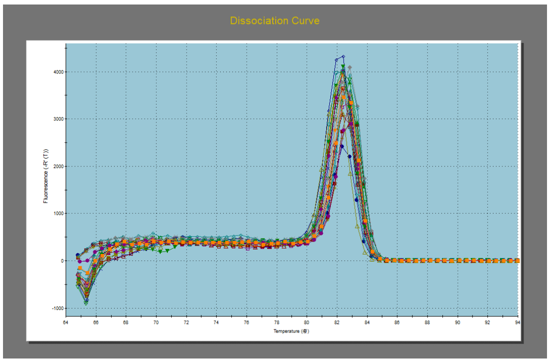

Bax 溶解曲线

BAX	Ct (dR)	actin	Ct (dR)	△CT	△△CT	相对表达量
	29.50		13.74	15.76	0.17	0.89
ck	28.69	ck	13.45	15.24	-0.35	1.27
	29.16		13.40	15.76	0.17	0.89
	30.79		13.61	17.18	1.59	0.33
si-NC	28.90	N	13.57	15.33	-0.26	1.19
	29.43		13.18	16.25	0.66	0.63
	26.81		13.09	13.72	-1.87	3.65
astaxanthin	27.90	E+G	13.64	14.26	-1.33	2.51
	28.23		13.33	14.90	-0.69	1.61
si-stat3	27.56	M+E	12.73	14.83	-0.76	1.69
	27.54		13.68	13.86	-1.73	3.31

	27.80		13.32	14.48	-1.11	2.15
	27.09		13.41	13.68	-1.91	3.75
combined	27.38	I+G	13.85	13.53	-2.06	4.16
	27.21		14.09	13.12	-2.47	5.53

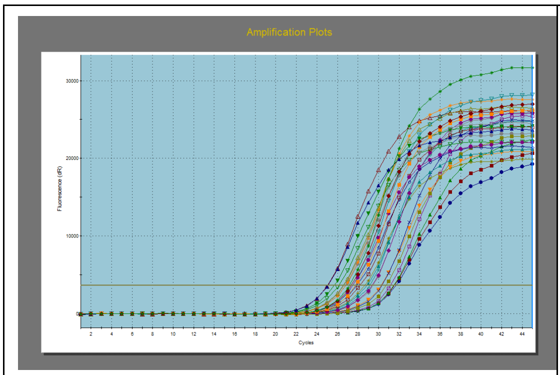

NF-KB 扩增曲线

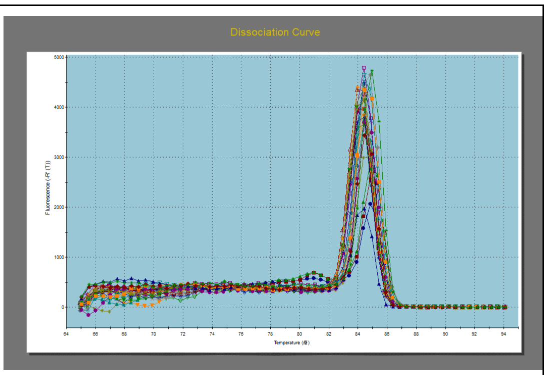

NF-KB 溶解曲线

NF-KB	Ct (dR)	actin	Ct (dR)	△CT	△△CT	相对表达量
	30.84		13.74	17.10	-0.60	1.52
ck	31.32	ck	13.45	17.87	0.17	0.89
	31.53		13.40	18.13	0.43	0.74
	31.21		13.61	17.60	-0.10	1.07
si-NC	31.91	N	13.57	18.34	0.64	0.64
	31.42		13.18	18.24	0.54	0.69
	33.01		14.11	18.90	1.20	0.44
astaxanthin	32.50	N+G	14.53	17.97	0.27	0.83
	33.12		14.47	18.65	0.95	0.52
	31.86		12.73	19.13	1.43	0.37
si-stat3	32.68	M+E	13.68	19.00	1.30	0.41
	32.14		13.32	18.82	1.12	0.46
	34.72		13.72	21.00	3.30	0.10
combined	34.31	I	14.55	19.76	2.06	0.24
	34.82		14.37	20.45	2.75	0.15

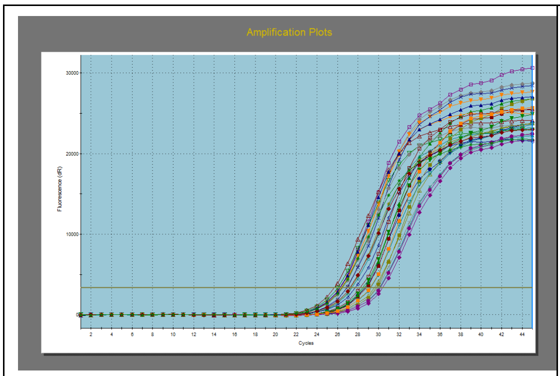

Casp3 扩增曲线

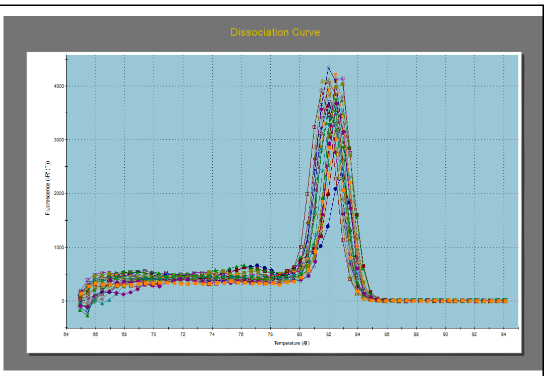

Casp3 溶解曲线

casp3	Ct (dR)	actin	Ct (dR)	△CT	△△CT	相对表达量
	28.52		13.74	14.78	-0.39	1.31
ck	28.93	ck	13.45	15.48	0.31	0.80
	28.64		13.40	15.24	0.07	0.95
	28.76		13.61	15.15	-0.02	1.01
si-NC	28.82	N	13.57	15.25	0.08	0.94
	28.66		13.18	15.48	0.31	0.80
	26.92		13.09	13.83	-1.34	2.53
astaxanthin	27.36	E+G	13.64	13.72	-1.45	2.73
	27.47		13.33	14.14	-1.03	2.04
	26.46		12.73	13.73	-1.44	2.71
si-stat3	27.78	M+E	13.68	14.10	-1.07	2.09
	28.26		13.32	14.94	-0.23	1.17
	26.31		13.41	12.90	-2.27	4.81
combined	27.26	I+G	13.85	13.41	-1.76	3.38
	26.49		14.09	12.40	-2.77	6.81

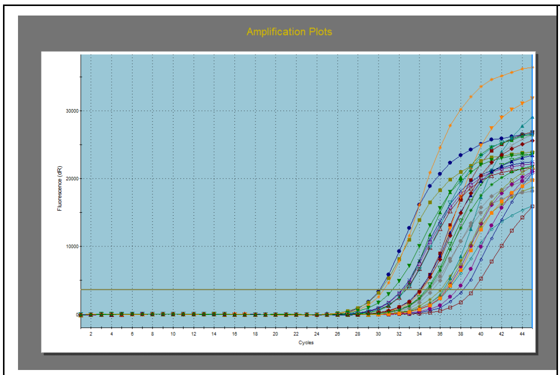

Casp9 扩增曲线

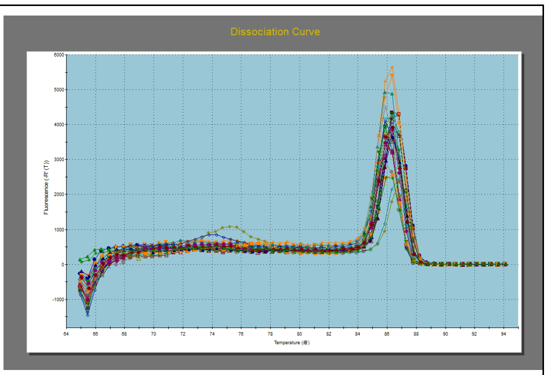

Casp9 溶解曲线

casp9	Ct (dR)	actin	Ct (dR)	△CT	△△CT	相对表达量
	34.76		13.74	21.02	-0.31	1.24
ck	35.08	ck	13.45	21.63	0.30	0.81
	34.74		13.40	21.34	0.01	0.99
	34.26		13.61	20.65	-0.68	1.60
si-NC	34.37	N	13.57	20.80	-0.53	1.44
	34.67		13.18	21.49	0.16	0.90
	33.42		13.09	20.33	-1.00	2.00
astaxanthin	33.90	E+G	13.64	20.26	-1.07	2.10
	33.49		13.33	20.16	-1.17	2.25
	34.04		13.41	20.63	-0.70	1.62
si-stat3	34.18	I+G	13.85	20.33	-1.00	2.00
	34.78		14.09	20.69	-0.64	1.56
	32.14		14.11	18.03	-3.30	9.85
combined	32.11	N+G	14.53	17.58	-3.75	13.45
	32.57		14.47	18.10	-3.23	9.38

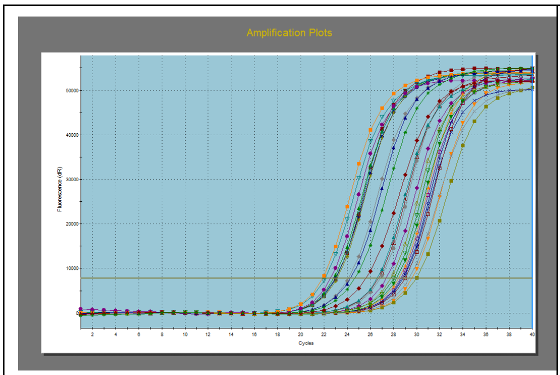

jak2 扩增曲线

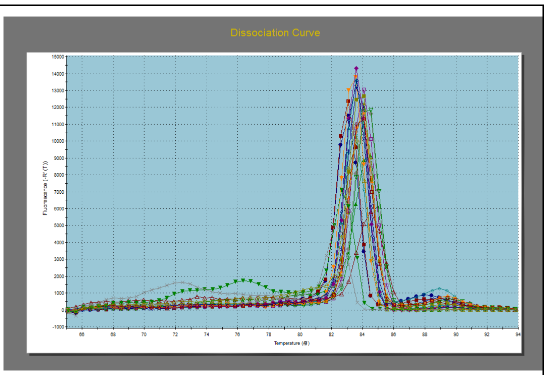

jak2 溶解曲线

jak2	Ct (dR)	actin	Ct (dR)	△CT	△△CT	相对表达量
	24.33		13.74	10.59	-0.44	1.35
ck	24.65	ck	13.45	11.20	0.17	0.89
	24.69		13.40	11.29	0.26	0.83
	24.14		13.61	10.53	-0.50	1.41
si-NC	23.15	N	13.57	9.58	-1.45	2.73
	23.03		13.18	9.85	-1.18	2.26
	28.30		14.11	14.19	3.16	0.11
astaxanthin	28.37	N+G	14.53	13.84	2.81	0.14
	28.78		14.47	14.31	3.28	0.10

	28.30		14.11	14.19	3.16	0.11
si-stat3	28.37	N+G	14.53	13.84	2.81	0.14
	28.78		14.47	14.31	3.28	0.10
	29.66		13.72	15.94	4.91	0.03
combined	29.62	I	14.55	15.07	4.04	0.06
	28.86		14.37	14.49	3.46	0.09

Animal expeiment

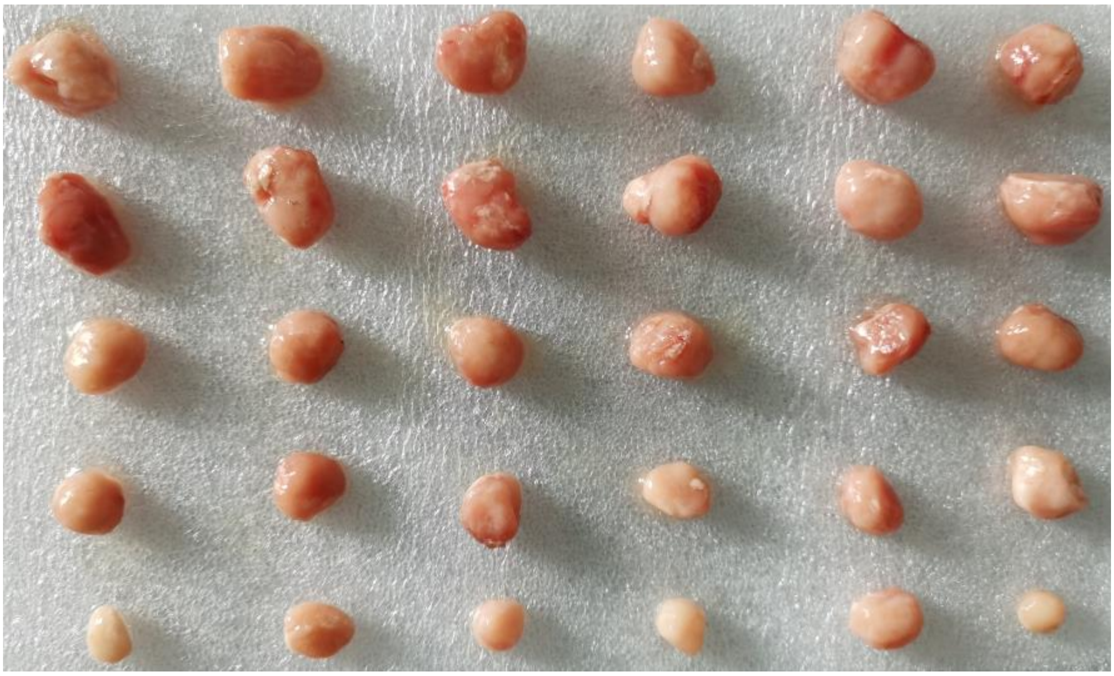

编号	质量 g	体积（长*宽*高）（mm）
CK1	1.98	16*12*12
CK2	1.96	18*13*11
CK3	1.93	14*12*10
CK4	1.48	13*10*10
CK5	2.29	16*13*12
CK6	1.60	14*14*10
si-NC1	1.78	18*10*10
si-NC2	2.20	16*11*10
si-NC3	2.01	14*13*12
si-NC4	2.27	16*14*14

si-NC5	1.80	15*13*12
si-NC6	2.58	18*14*12
si-Stat3-1	1.20	12*11*8
si-Stat3-2	1.28	10*9*9
si-Stat3-3	1.12	10*9*8
si-Stat3-4	1.78	12*11*10
si-Stat3-5	1.23	13*9*8
si-Stat3-6	1.21	13*8*8
astaxanthin-1	0.92	9*8*7
astaxanthin-2	1.02	9*7*7
astaxanthin-3	1.10	10*8*8
astaxanthin-4	0.81	9*8*6
astaxanthin-5	0.88	10*8*7
astaxanthin-6	1.19	12*10*8
Astaxanthin+si-STAT3-1	0.33	5*4*3
Astaxanthin+si-STAT3-2	0.66	8*6*5
Astaxanthin+si-STAT3-3	0.43	5*5*4
Astaxanthin+si-STAT3-4	0.45	6*4*4
Astaxanthin+si-STAT3-5	0.91	9*8*6
Astaxanthin+si-STAT3-6	0.38	4*4*3

Western blotting experiments

The original unmodified blots of the Western blot experiments. In the Western blot images presented in this study, it is important to note that some of the blots were cut based on the size of the protein of interest and the positions indicated by the protein size markers as a cost-saving measure to optimize the use of antibodies and focus on specific bands of interest to minimize antibody use. Additionally, it is important to note that in certain cases, the edges of PVDF membranes may not be clearly visible due to pixel limitations, resulting in edges blending with the background color.

1. **β-actin** (Below are three images with different exposure levels).

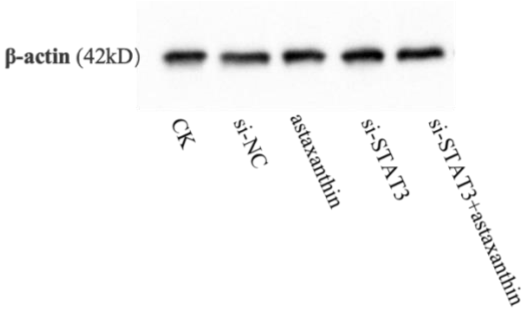

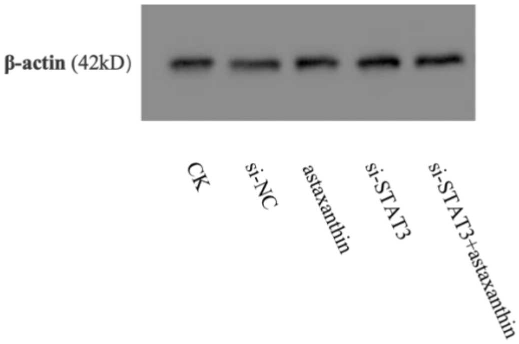

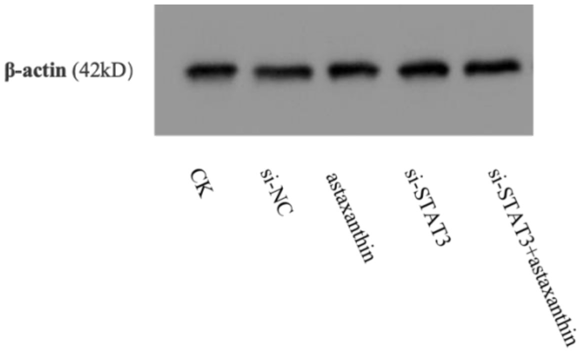

2. NK-Kβp65 (Below are three images with different exposure levels).

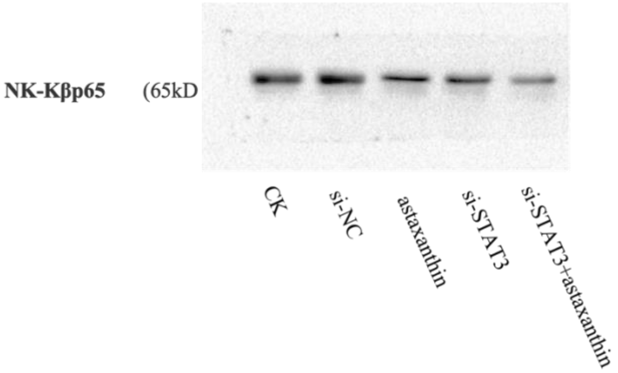

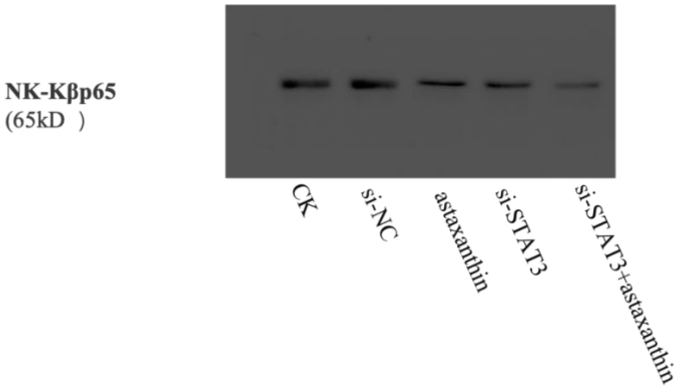

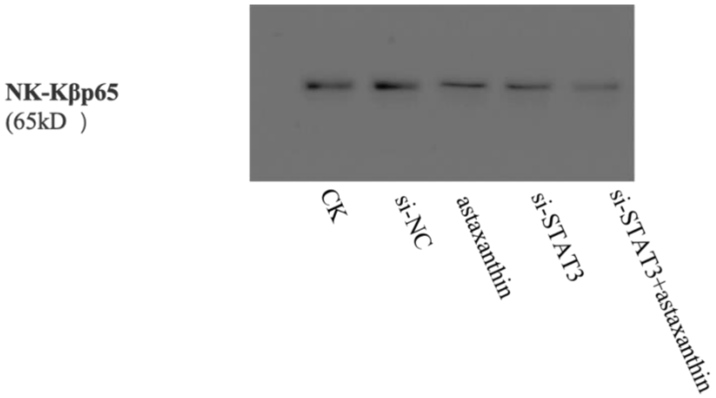

3. Bcl-2 (Below are three images with different exposure levels).

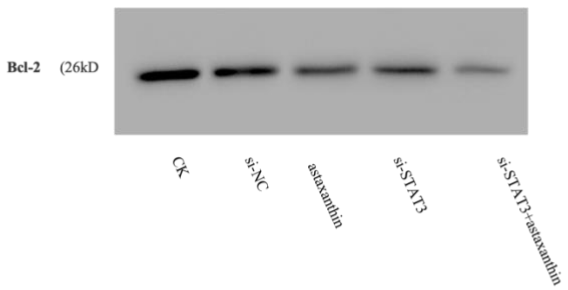

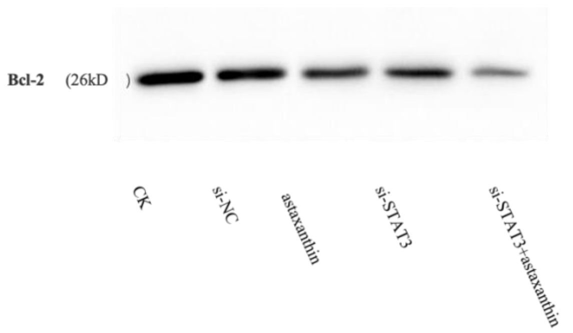

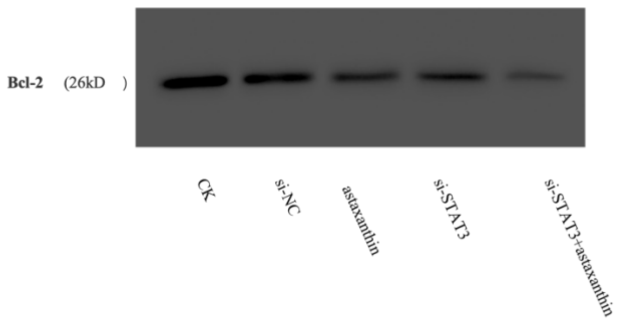

4. **Caspase3** (Below are three images with different exposure levels).

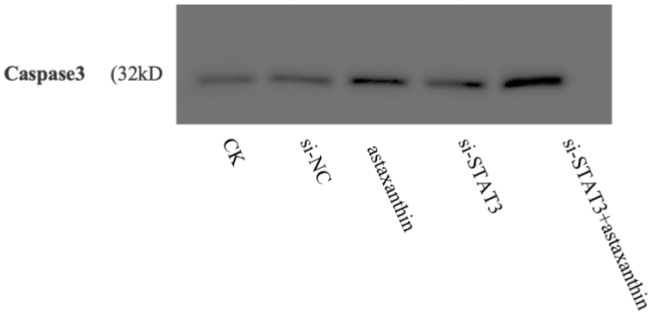

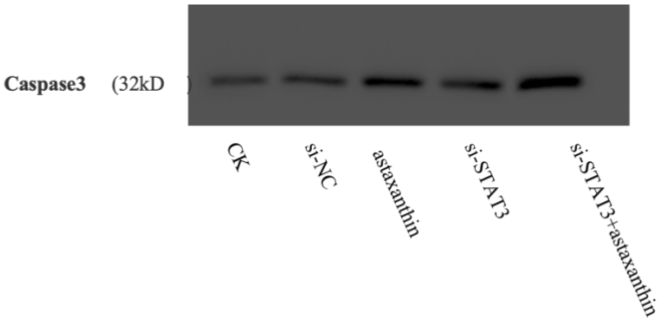

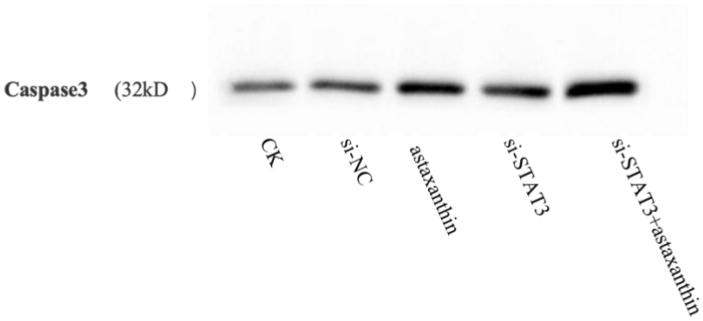

5. **Caspase9** (Below are three images with different exposure levels).

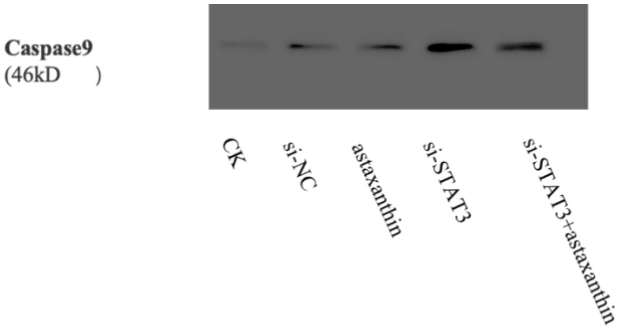

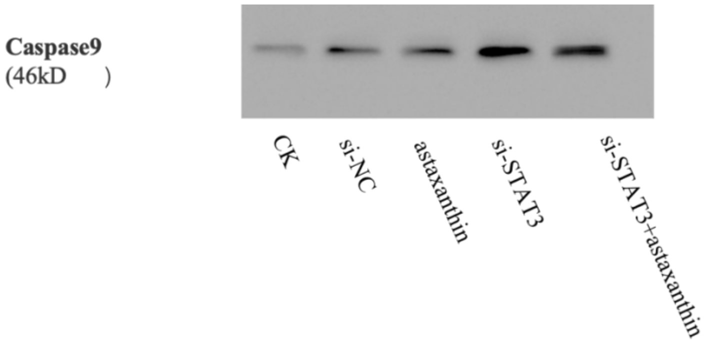

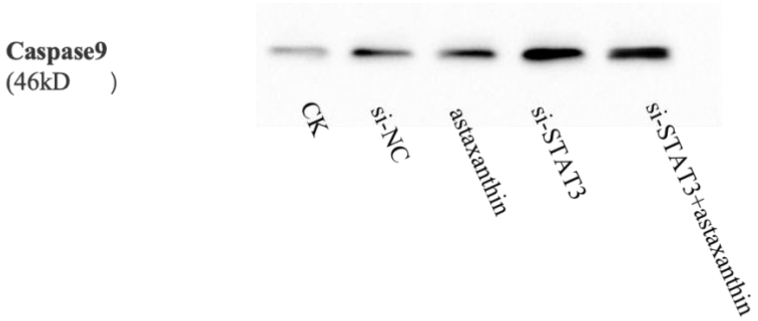

6. JAK-2 (Below are three images with different exposure levels).

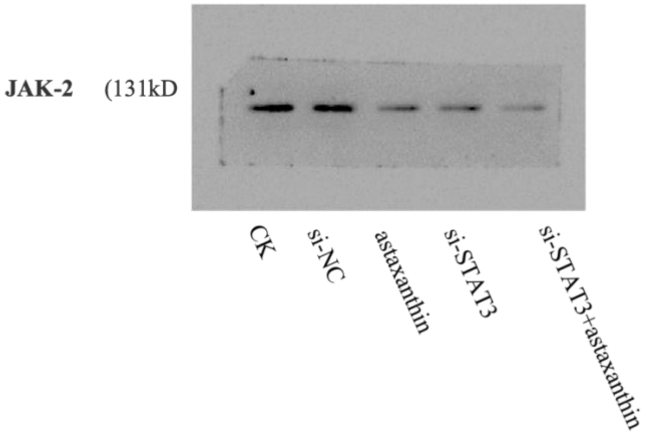

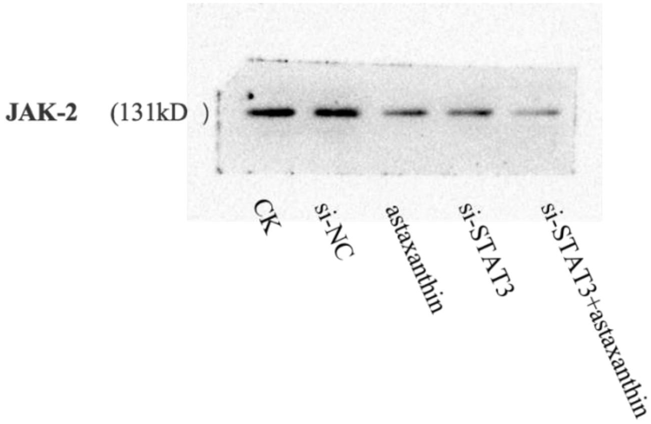

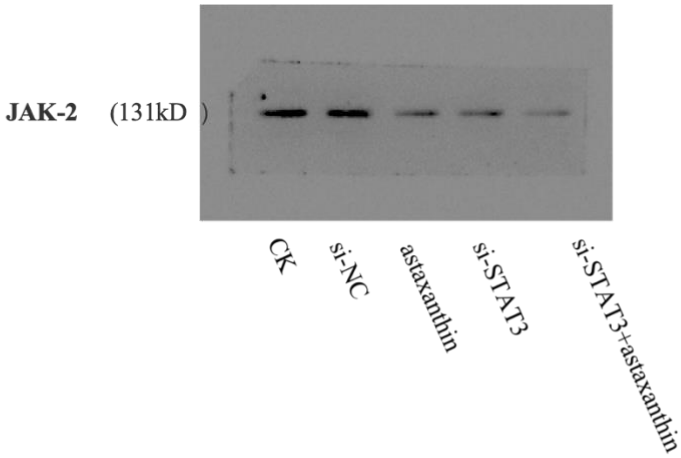

7. **BAX** (Below are three images with different exposure levels).

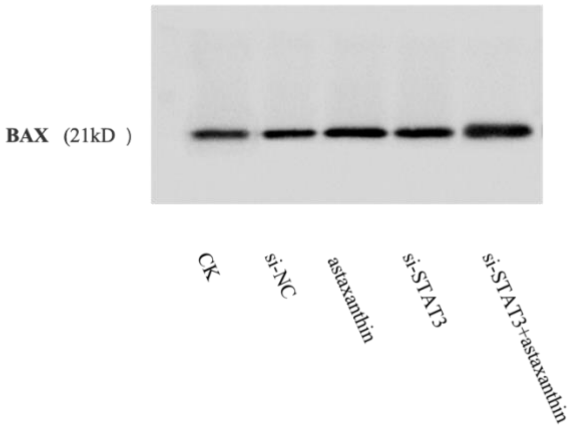

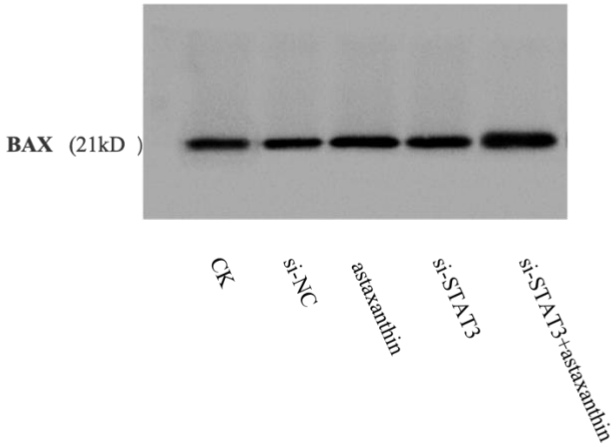

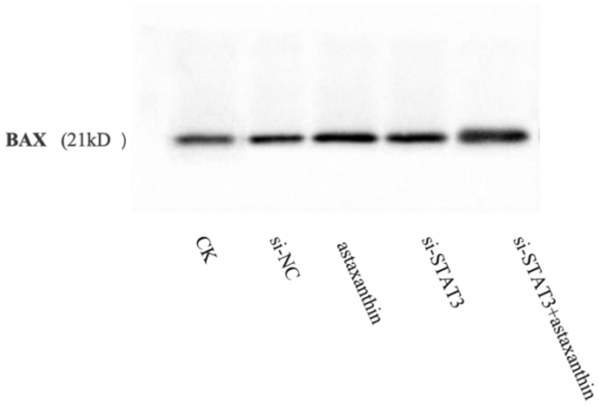

8. Stat3 (Below are three images with different exposure levels).

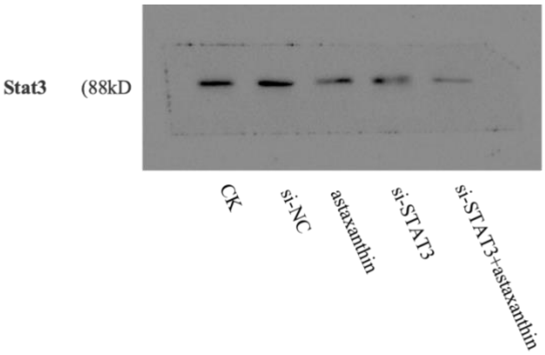

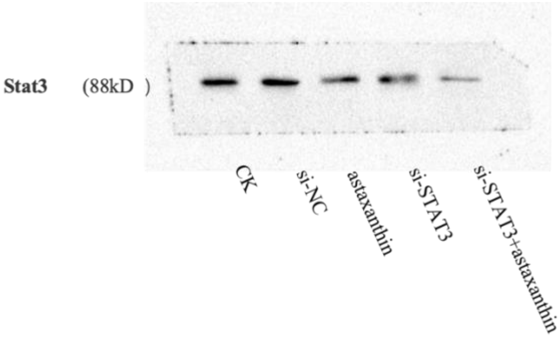

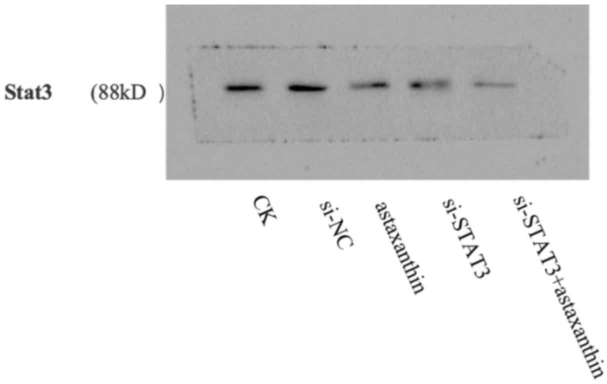

Tunel experiment

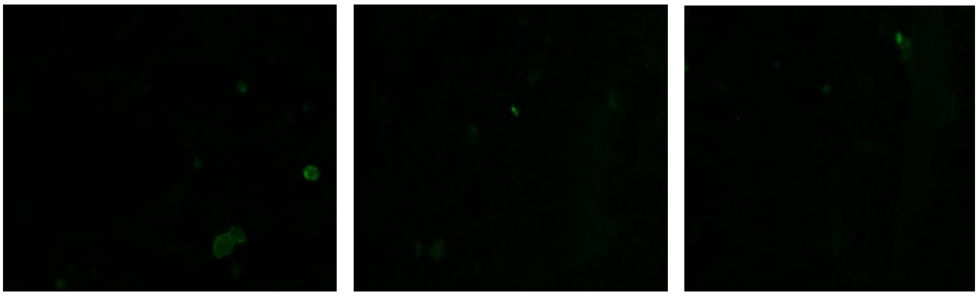

CK-1 CK-2 CK-3

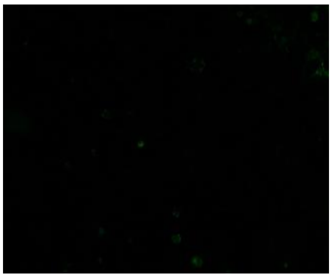
si-NC-1

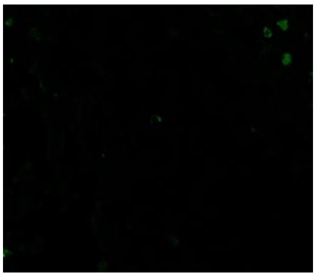
si-NC-2

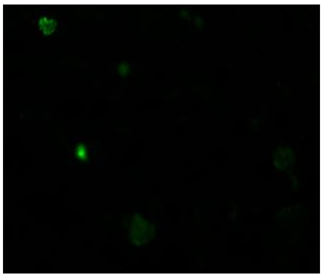
si-NC-3

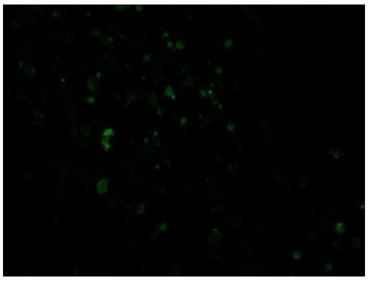
Stat3-1

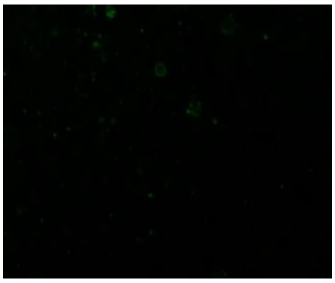
Stat3-2

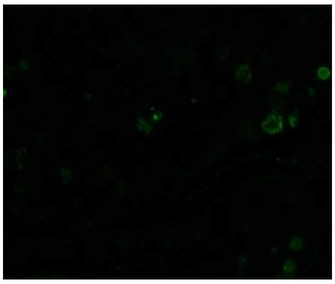
Stat3-3

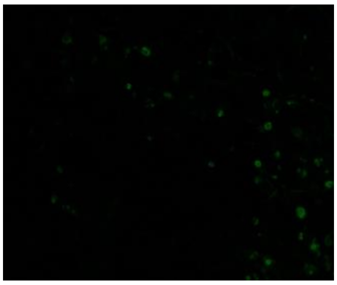
si-stat3-1

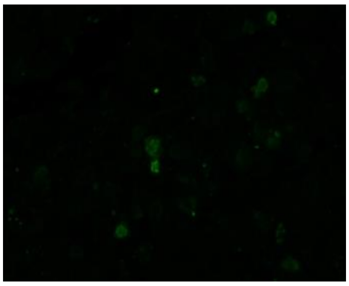
si-stat3-2

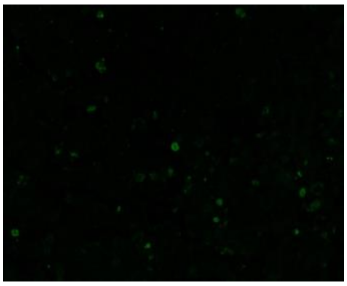
si-stat3-3

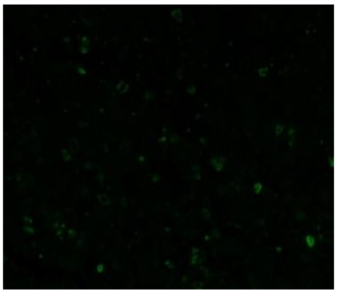
Combined-1

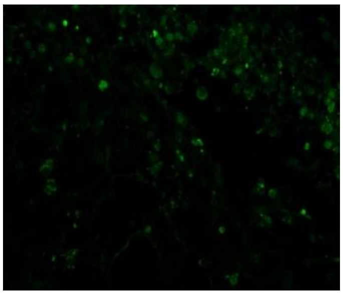
Combined-2

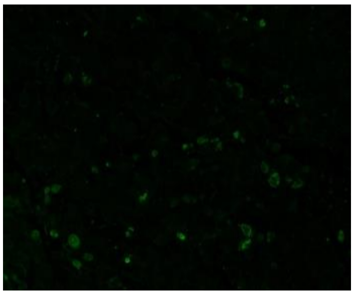
Combined-3
